# Lipid-II Independent Antimicrobial Mechanism of Nisin Depends On Its Crowding And Degree Of Oligomerization

**DOI:** 10.1038/srep37908

**Published:** 2016-11-29

**Authors:** Ashutosh Prince, Padmani Sandhu, Pankaj Kumar, Eva Dash, Shingarika Sharma, Manoranjan Arakha, Suman Jha, Yusuf Akhter, Mohammed Saleem

**Affiliations:** 1 Department of Life Sciences, National Institute of Technology, Rourkela, India; 2 Centre for Computational Biology and Bioinformatics, School of Life Sciences, Central University of Himachal Pradesh, India

## Abstract

Nisin inhibits bacterial growth by generating pores in cell membrane and interrupting cell-wall biosynthesis through specific lipid II interaction. However, the role of the hinge region and C-terminus residues of the peptide in antibacterial action of nisin is largely unknown. Here, using molecular dynamics simulations and experimental approach, we report that at high concentration regimes of nisin, interaction with phospholipids may equally deform the bacterial cell membranes even under significantly varying amounts of lipid-II. Membrane thinning, destabilization and decrease in lipid density depend on the degree of oligomerization of nisin. Growth kinetics of *Bacillus subtilis* and *Escherichia coli* interestingly show recovery by extended lag phase under low concentrations of nisin treatment while high concentrations of nisin caused decrease in cell viability as recorded by striking reduction in membrane potential and surface area. The significant changes in the dipole potential and fluorescence anisotropy were observed in negatively charged membranes in the absence of lipid-II with increasing concentration of nisin. The identical correlation of cell viability, membrane potential dissipation and morphology with the concentration regime of nisin, in both *Bacillus subtilis* (lipid II rich) and *Escherichia coli* (lipid II impoverished), hints at a non-specific physical mechanism where degree of membrane deformation depends on degree of crowding and oligomerization of nisin.

Excessive use of antibiotics for treatment has led to parallel evolution of bacteria with multidrug resistance^1^. Antimicrobial peptides (AMP) present an effective mode of combating pathogenic bacteria by destabilizing the bacterial cell membrane^2^. AMPs are known to deform bacterial cell membrane by various mechanisms: a) detergent-like solubilization upon aggregation parallel to the membrane plane^3^; b) the formation of toroidal pores or barrel-stave upon membrane-spanning perpendicular oligomeric assembly of the peptide^4^,^5^,^6^. Only very few of the AMPs are allowed to be used as a food preservative or an antibiotic in health care and one such unique case that has attracted significant attention in the recent times is the lantibiotic nisin^7^.

Nisin, a 34 amino acid residue long cationic peptide, belongs to class I pore-forming bacteriocins and displays a unique pore-forming activity against bacteria which is known to be enhanced in the presence of lipid II^8^,^9^. First structural insights of the nisin-lipid II complex revealed that nisin binds to lipid II with the two lanthionine rings at the N-terminus, forming a pyrophosphate cage around the head-group of lipid II^10^. Initially the peptide lies parallel to the membrane plane and the insertion of the C-terminus in a transmembrane orientation, facilitated by the flexible hinge region, eventually leads to stable pore formation^8^,^11^,^12^. In addition to generation of pores, nisin is known to inhibit cell wall biosynthesis by interrupting the peptidoglycan production^9^. An alternative mechanism of bactericidal action of nisin involves the segregation and loss of lipid II^13^.

Pore formation is believed to cause rapid dissipation of transmembrane electrostatic potential resulting in membrane permeabilisation and rapid bacterial cell death^8^,^14^. On the contrary, membrane permeabilisation was found to trigger metabolic deregulation of bacterial division leading to accelerated cell division, thereby, suggesting late cell death^15^. The mechanism of membrane deformation during bacterial cell death upon antibiotic treatment thus remains unclear, primarily, because of observations based on different model bacteria, experimental approaches used and time-scale of growth kinetics. Moreover, conventional minimum inhibitory concentrations (MIC) derived from optical density data in cultures may not fully reflect the diverse states of bacteria in terms of viability^16^,^17^,^18^. The mechanism of pore formation mediated by specific interaction between nisin and lipid II is well established^10^,^19^,^20^. However, lipid II is present in relatively small amounts in bacteria^21^ and given the lifetime of the pore^12^, the role of nisin interaction with other phospholipids becomes critical.

We therefore investigated the interaction of hinge region and C-terminal residues of nisin with phospholipid bilayers by MD simulations for the first time, to our knowledge, and found that nisin at high concentration regimes can strongly deform the membrane even in the absence of lipid II. The rate of membrane deformation was dependent on the oligomeric state of nisin. We then experimentally validated the MD observations by showing that nisin equally deforms both *B. subtilis* and *E. coli* in a concentration dependent manner. At molecular level, dipole potential and anisotropy measurements in reconstituted membranes strongly suggest nisin interaction with negatively charged membrane even in the absence of lipid II. These findings demonstrate that nisin exposure in a collective population of bacteria is heterogeneous; therefore, the time-scale of death of the bacteria depends on the surface bound density of nisin. We propose a non-specific physico-chemical model of nisin action (that may be lipid II mediated) that drives membrane deformation dependent on its membrane surface bound density by showing the correlation between nisin concentration, membrane destabilization (surface/dipole potential, fluorescence anisotropy and intensity) and cell viability. This study will provide a complementary perspective to the existing molecular understanding of nisin mediated antibacterial activity.

## Results

### Nisin oligomerization causes increased water permeation by triggering acyl-chain disorder and lowered lipid packing density

**Figure 1:**
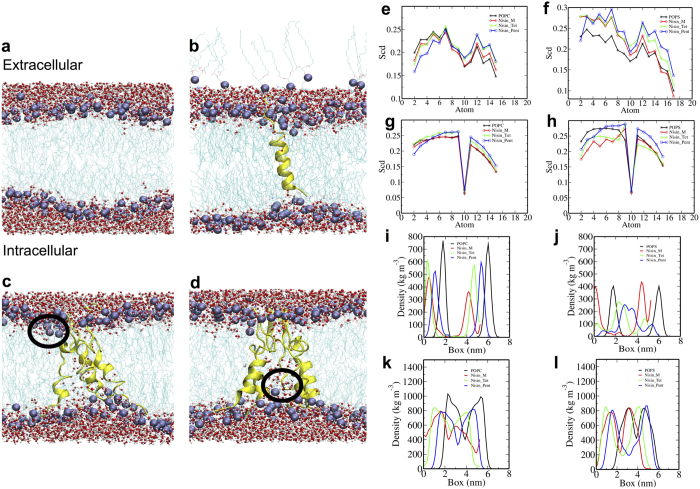
Nisin oligomerization leads to increased membrane permeability, curvature and lowering in lipid packing density. (**a**) No water defects in the lipid bilayer were observed in the absence of nisin peptides. (**b**) No water defects were observed in the lipid bilayer on insertion of nisin monomer but water molecules centered on the peptide molecule, nisin peptide is shown in yellow colour in cartoon. (**c**) Water defects in the lipid bilayer on insertion of nisin tetramer (encircled). (**d**) Water defects in lipid bilayer on insertion of nisin pentamer (encircled). Lipid membrane is shown in cyan colour in lines and lipid head-groups in ice blue colour in vdw model, water molecules in red and white colour in cpk colour scheme. Panels (**e–h**) show deuterium order parameter of sn2 and sn1 chain of POPC and POPS. X-axis represents the number of atoms while the Y-axis represent order parameter (Scd). (**e**) sn2 chain of the POPC molecules in the absence and presence of nisin peptide immersed in POPC + POPE + POPS bilayer, (**f**) sn2 chain of POPS molecules in the absence and presence of nisin peptide immersed in POPC + POPE + POPS bilayer, (**g**) sn1 chain of POPC molecules in the absence and presence of nisin immersed in POPC + POPE + POPS bilayer, (**h**) sn1 chain of POPS molecules in the absence and presence of nisin immersed in POPC + POPE + POPS bilayer. Panels (**i–l**) show partial densities of lipid head groups and tails. X-axis represents the box dimensions along the Z – direction of the lipid bilayer in nm, while Y-axis represents the density in Kg m^−3^, (**i**) POPC lipid head-groups in POPC + POPE + POPS bilayer without and after insertion of nisin monomer and oligomers, (**j**) POPS lipid head-groups in POPC + POPE + POPS bilayer before and after insertion of nisin monomer and oligomers, (**k**) POPC lipid tail-groups in POPC + POPE + POPS bilayer before and after insertion of nisin monomer and oligomers, **(l)** POPS lipid tail-groups in POPC + POPE + POPS bilayer before and after insertion of nisin monomer and oligomers.

Insertion of nisin in the membrane was found to trigger accumulation of water molecules near the insertion point of the peptide unlike the pure POPC (1-palmitoyl-2-oleoyl-sn-glycero-3-phosphocholine) bilayer where there was no such permeation (Fig. 1a and b–d). The degree of water permeation increased with higher oligomerization state of nisin (i.e., monomer <tetramer <pentamer assembly) (Fig. 1b–d). The deuterium order parameter is the measurement of the orientation movability of the bond between C and D (C-D), where C is the carbon atom of acyl chain and D is the deuterium. It is referred as an indication of order or disorderness of the acyl chains of lipid molecules. The deuterium order parameter analysis for the mixed bilayer showed that the sn2 acyl chain of POPC and POPS (1-palmitoyl-2-oleoyl-sn-glycero-3-phospho-L-serine) molecules were having strongest deviations in the case of nisin pentamer-lipid complex (Fig. 1e and f). The deviations also showed an increase in the order parameter for carbon positions towards the end of the sn1 acyl chain of POPC and POPS molecules from nisin-pentamer-mixed lipid bilayer complex as compared to the POPC and POPS from mixed lipid bilayer without any nisin molecule (Fig. 1g and h). We then analyzed the changes in density of the membrane for the lipid head-groups and tails. It can be observed that for mixed bilayer the head-groups were symmetrically arranged at upper and lower leaflets with a density of 620 Kg m^−3^ and occupying maximum dimensions of 2 nm in the absence of nisin (Fig. 1i and j). Insertion of monomer nisin led to the increased density of head-groups up to 710 Kg m^−3^ for both leaflets, however, there was a decrease in the density of head-groups after the insertion of tetramer and pentamer. The analysis of head-group densities for POPC and POPS molecules showed that the density of head-groups was decreased for POPC molecules but remained evenly distributed (Fig. 1i). The decreased head-group density shows accumulation and increase in the centre of Z-Plane of mixed lipid bilayer indicating shift in the position of lipid head-groups POPS molecules toward the middle of membrane (Fig. 1j). Further, the insertion of pentameric nisin in the mixed lipid bilayer was found to cause strongest decrease in the density of tail-groups. Interestingly, a shift was observed toward the periphery of the lipid bilayer plane as compared to their even distribution in normal bilayer without any peptide indicating the flipping of lipid tails in the presence of nisin (Fig. 1k–l).

### Nisin prolongs bacterial lag phase in a concentration dependent manner

**Figure 2:**
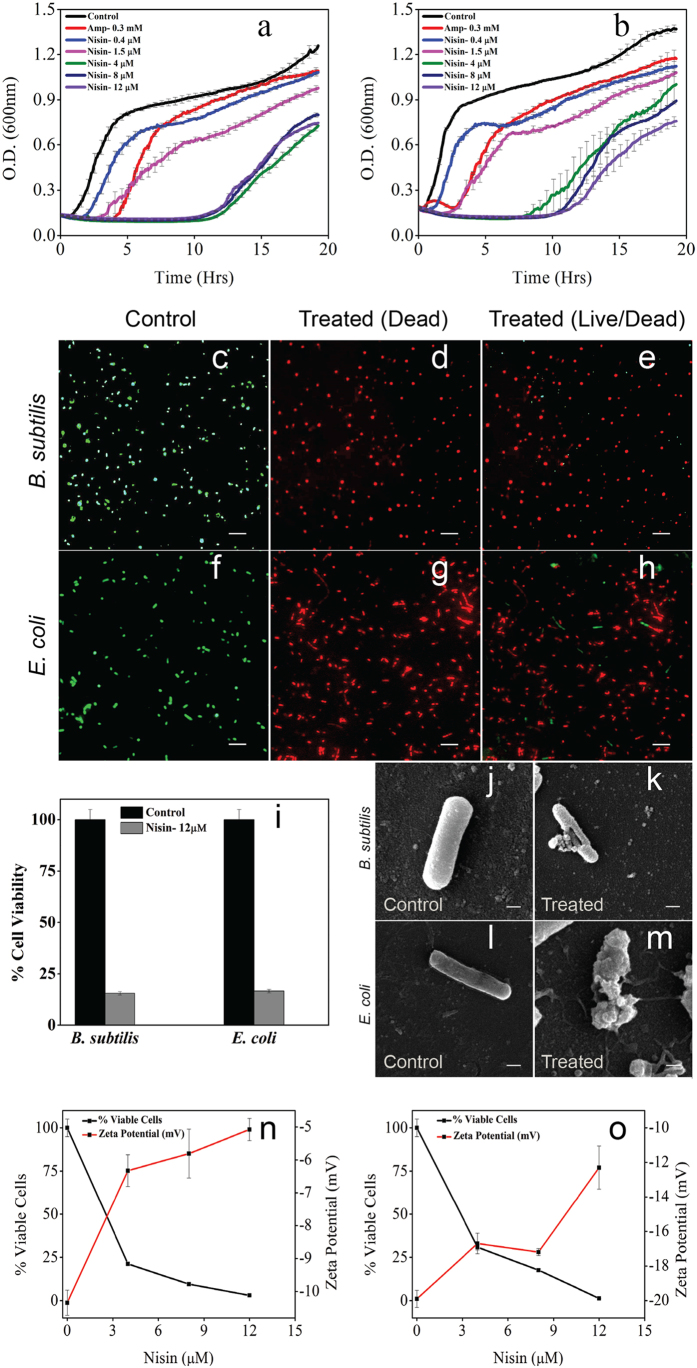
Cell viability of *Bacillus subtilis and Escherichia coli* is dependent on concentration of nisin. (**a**,**b**) Increasing concentrations of nisin triggers an extended-lag phase in growth kinetics of bacteria (a - *B. subtilis*; b - *E. coli*). In each case black curve represents positive control condition, which lacks nisin treatment. Red curve is a negative control for ampicillin treatment (0.3 mM) to show ampicillin resistance of bacteria. Remaining curves represent nisin treatment of 0.4, 1.5, 4, 8, and 12 μM concentrations, respectively. (Time scale 20 hrs for all experiments, no. of independent experiments, n = 3) (**c**,**f**) Untreated bacterial cells with stable membranes show green fluorescence (c - *B. subtilis*; f - *E. coli*). (**d**,**g**) Bacterial cells treated with lethal dose of nisin fluoresce red due to deformed membranes (d - *B. subtilis*; g - *E. coli*). (**e**,**h**) Merged image of viable (green) and dead bacteria (red) (e - *B. subtilis*; h - *E. coli*) showing relative populations of viable and dead cells upon treatment with lethal dose of nisin. (**i**) Histogram representing percentage cell viability (n = 125 cells) (**j**,**k**,**l**,**m**) Electron micrograph showing the morphology of bacteria treated with nisin (**h**,**j** - untreated *B. subtilis* and *E. coli*; **j**,**k** nisin treated *B. subtilis* and *E. coli*). (**n**,**o**) Plots showing correlation between the bacterial membrane surface potential, cell viability and concentration of nisin. Black line represents cell viability of both strains and red line denotes membrane Zeta potential at different concentrations of nisin with mean and standard error (**n**) - *B. subtilis*; (**o**) - *E. coli*). (The scale bars −20 μm for fluorescence images; 500 nm for electron micrographs).

To study the effect of nisin’s pore formation on the bacterial cell membrane during various growth phases (particularly lag and exponential), we investigated the morphological changes that take place in *Bacillus subtilis* and *E. coli* upon *in vivo* exposure to increasing concentrations of nisin. Addition of increasing concentrations of nisin lead to prolongation in the lag phase as well as inhibition of overall growth measured over 20 hours, as shown in Fig. 2a (*B. subtilis*) and Fig. 2b (*E. coli*). The minimum inhibitory concentration of Nisin for *B. subtilis* and *E. coli* were found to be 3.06 ± 1.1 μM and 3.07 ± 1.5 μM, respectively. In order to measure the relative effectiveness of nisin we compared growth kinetics of nisin-treated bacteria to that of bacteria treated with 0.3 mM of Ampicillin, to which both the bacterial strains are resistant. Lower concentrations of nisin upto 1.5 μM showed minimal inhibition of growth. Higher concentrations (i.e., 4 μM, 8 μM and 12 μM) not only significantly prolonged the lag phase but also drastically inhibited the overall growth in both the bacteria. The lag phase was prolonged by ~11–12 hours in both *B. subtilis* and *E. coli.* While concentrations of 4–12 μM of nisin were more or less equally effective in inhibiting the growth in case of *B. subtilis* (Fig. 2a), maximal growth inhibition for *E. coli* was induced by 12 μM of nisin (Fig. 2b). The higher concentration of nisin leads to complete inhibition of bacterial growth evident from the electron microscopy and fluorescence cell viability assay described later. Most surprisingly, we observed that higher concentrations of nisin (12 μM) was able to efficiently inhibit the growth in the gram negative *E. coli* too challenging the general notion that nisin can only target gram negative bacteria in the presence of EDTA or heat shock.

### Fluorescence based quantification of viability of bacterial cells

We then investigated bacterial cell viability upon treatment with 12 μM of nisin to correlate the obtained OD to that of the state of bacterial population (i.e., if it resulted in death or prolonged growth inhibition within stationary phase). To visualize whether the bacterial populations were in a state of prolonged growth inhibition or dead, we carried out LIVE/DEAD BacLight Fluorescent assay. In this assay the viable bacterial cells having an intact membrane are visualized green by interaction of Syto9 fluorescent dye. On the contrary, non-viable bacterial cells with porous membranes enable the propidium iodide to enter and interact with the bacterial nucleic acids giving rise to red fluorescence. It was observed that *B. subtilis* (Fig. 2c–e) and *E. coli* (Fig. 2f–h) not treated with nisin are stained green suggesting they have intact cell membranes while nisin (12 μM) treated *B. subtilis and E. coli* results in bacterial cell death due to the membrane disruptions. The merged images show corresponding proportions of viable (green) and non-viable (red) populations of bacteria. About 90% bacterial cell deaths occur at a concentration of 12 μM of nisin in both *B. subtilis* and *E.coli* as can be inferred from the histogram (Fig. 2i). Electron microscopy of bacterial surface morphology also revealed striking membrane deformation induced by lethal dose of nisin triggering death in both *B. subtilis* (Fig. 2j–k) and *E. coli* (Fig. 2l–m).

### Nisin causes concentration dependent dissipation of membrane potential

As fluorescence based cell viability assay hints at the deformation of the bacterial cell membrane. Therefore, we first investigated the effect of nisin on bacterial membrane surface potential. The native membrane surface potential of *B. subtilis* and *E. coli* were found to be −11.6 mV and −20 mV respectively. Nisin induced neutralization of membrane surface potential resulted in gradual increase upto −5 mV in *B. subtilis* and −12 mV in case of *E. coli*, with increasing concentration upto 12 μM as shown in Fig. 2n and o. Further, in order to estimate the fold change in the bacterial cell viability due to the treatment with increasing concentrations of Nisin, we determined the colony-forming unit per mL (cfu/mL) with respect to Nisin concentration. MBC was found to be 12 μM for both *B. subtilis* and *E. coli*.

The correlation between the cell viability and the membrane potential also suggested the possible deformation of the membrane by the nisin interaction.

### Effect of nisin concentration on the morphology of bacterial membrane

**Figure 3:**
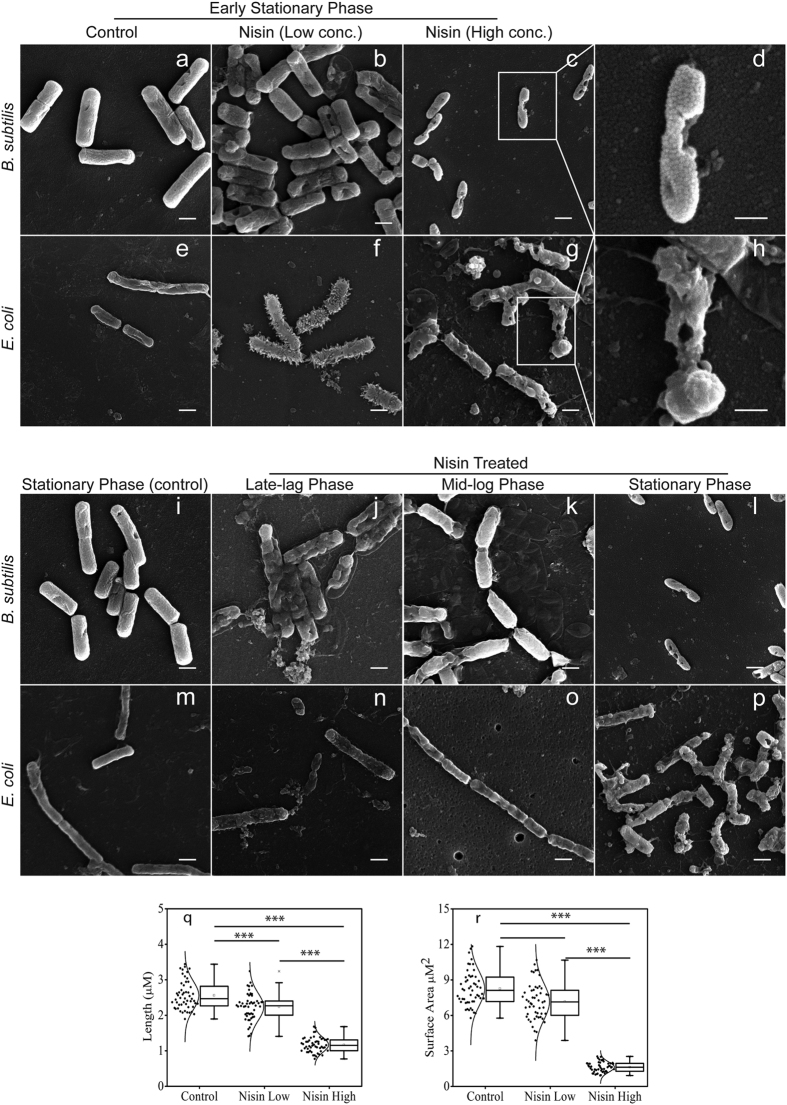
Electron microscopy of the bacterial surface morphological changes triggered by nisin. Electron micrographs showing membrane morphology of *B. subtilis* and *E. coli* under following conditions: (**a**,**e**) without treatment of nisin, (**b**,**f**) 0.4 μM nisin-treatment and (**c**,**g**) 12 μM nisin-treatment. Zoomed in electron micrographs are showing strikingly deformed membrane with pores in *B. subtilis* (**d**) and excessive blebbing in *E. coli* (**h**). Electron micrographs showing membrane morphology of *B. subtilis* and *E. coli* under following growth phase and conditions: (**i**,**m**) stationary phase without any nisin-treatment (control); (**j**,**n**) nisin-treated (12 μM) bacteria in late lag phase show minor membrane deformations with little effect on size and shape; (**k**,**o**) nisin-treated (12 μM) bacteria in mid-exponential phase show chain like structures with multiple septa; (**l**,**p**) nisin-treated (12 μM) bacteria in stationary phase show drastic reduction in size, significant membrane disruption and pores formed in case of *B. subtilis* **(l**). Excessive membrane damage, pores, and blebbings were found in case of *E. coli* (**p**). (scale bars for electron micrographs, 1 μm; for zoomed images scale bar is 500 nm) (**q**,**r**) The box plot showing the effect of nisin concentration on the length and surface area of *B. subtilis*. Box plot shows the median, lower/upper quartiles and maximum/minimum values. (***P < 0.0001 in t-test).

We then investigated the morphological changes observed in bacteria reaching early stationary phase as a result of nisin induced membrane deformation that is ultimately leading to bacterial cell death. We used a concentration of 0.4 μM of nisin to compare the least effective concentration and 12 μM that were found to cause maximum effect established from cell viability assessed through growth kinetics and LIVE/DEAD fluorescence assay. Electron Microscopy of *B. subtilis* treated with nisin showed a concentration dependent reduction in the size in stationary phase. While the *B. subtilis* treated with 0.4 μM were roughly of the same size as untreated bacteria in stationary phase, however, surface roughness was found to increase. Strikingly, a five-fold reduction in the surface area of *B. subtilis* as well as further increase in surface roughness was observed when treated with 12 μM of nisin (Fig. 3a–d and q–r); For surface roughness analysis please see Supplementary Table S1). The otherwise rod shaped *B. subtilis* had undergone significant membrane deformation with tapered ends. Interestingly, *E. coli* treated with 0.4 μM nisin appeared to have generated numerous spike-like membrane protrusions on surface, without any significant changes in the size. However, higher concentration of nisin (12 μM) drastically deformed the membrane, as *E. coli* appeared to have generated numerous bleb-like membrane protrusions that resulted in loss of intact shape and size (Fig. 3e–h and see Supplementary Table S1 for surface roughness variations).

### Nisin induced morphological changes during different phases of bacterial growth

We further asked whether nisin predominantly affects lag, exponential or stationary phases or acts all along the bacterial growth phases. In order to address this we analyzed the morphological features of membrane surface of nisin (12 μM) treated bacteria fixed in late lag, mid-log, and stationary phase. *B. subtilis* showed uneven deformed surface morphology with condensed bleb-like membrane protrusions having low curvature in late lag phase as seen in Fig. 3j. *B. subtilis* were found to have an increased membrane roughness and occasional debris from dead cells suggesting the immediate action of nisin in a certain proportion of bacteria. *E. coli* also showed a similar distortion of morphology, however, with a larger number of smaller bleb-like membrane protrusions. Occasional throwing of cellular debris by dead cells was also observed (Fig. 3m). Interestingly, both *B. subtilis* and *E. coli* in mid-exponential phase were found to have recovered from bleb-like protrusions, however, surface roughness increased further compared to that in the late lag phase (Fig. 3k and o and Supplementary Table S2). Likewise, both were found to have formed a string of cells suggesting accelerated cell division. In stationary phase both bacterial strains were found to have highly deformed membrane. While *B. subtilis* showed numerous pores and membrane region with completely ruptured membranes, *E. coli* surface showed intense blebbing. *B. subtilis* were found to have been drastically reduced in size whereas *E. coli* showed deformed membrane surface with blebs and tubules, both cases resulting in cell death supporting the fluorescence based cell viability observations (Fig. 3l and p).

### Fluorescence anisotropy and dipole potential show DOPS to be the major interaction partner of nisin

**Figure 4:**
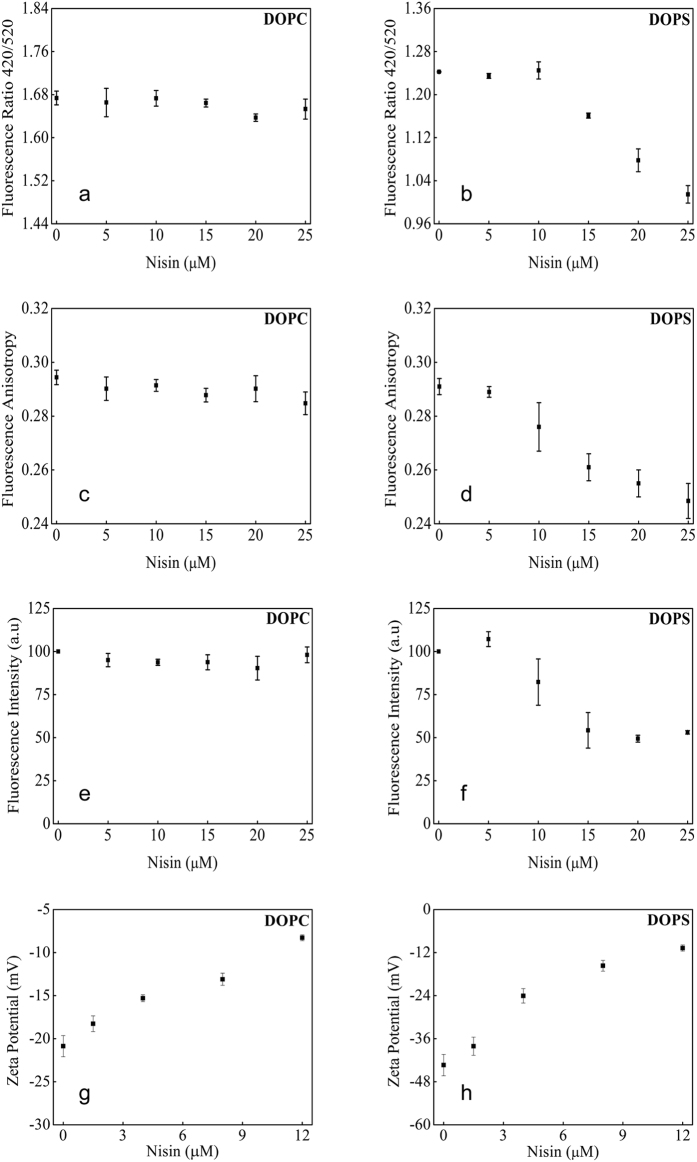
Changes in intrinsic membrane potentials and fluorescence anisotropy of pure phospholipid membranes by nisin. The dipole potential measured by dual wavelength ratiometric method using di-8-ANEPPS as probe shows - (**a**) no change in presence of nisin in DOPC vesicle membranes while, (**b**) linear decrease above 10 μM concentration of nisin in DOPS vesicles. Fluorescence anisotropy measured at an excitation wavelength of 460 nm and that of emission at 560 nm - (**c**) DOPC membranes show no significant change in anisotropy of membrane interfacial region due to nisin and (**d**) there is a uniform change in DOPS with increasing concentration of nisin. Fluorescence intensity of dye (**e**) not changed in case of DOPC but (**f**) reduced in DOPS membranes. Anisotropy and intensity were measured using excitation wavelength of 460 nm and emission was monitored at 560 nm. (**g**,**h**) show dissipation of membrane surface potential due to increasing concentration of nisin in DOPC and DOPS bilayers, respectively. Data points shown are the means ± S.E. of at least three independent measurements.

The dipole potential, measured by potentiometric probe, di-8-ANEPPS, did not change significantly for DOPC in presence of nisin (Fig. 4a). Lower concentration of nisin was found to have no effect on the dipole potential of DOPS membranes. However, the dipole potential was reduced by ~15% as nisin concentration was increased from 10 μM to 25 μM (Fig. 4b). Similar results were obtained in fluorescence anisotropy and intensity measurements. DOPC membranes did not show any significant decrease in the anisotropy (Fig. 4c). Fluorescence anisotropy in DOPS membranes was found to decrease almost linearly (uniformly) with increasing nisin concentration (Fig. 4d) DOPS membranes showed 47% decrease in relative intensity of di-8-ANEPPS in the presence of nisin as compared to no effect on relative intensity of dye in DOPC membranes (Fig. 4e–f). Further, the membrane surface potential of DOPC and DOPS membranes, were drastically neutralized upon nisin interaction in the absence of lipid II (Fig. 4g–h) suggesting significant non-specific interaction of nisin with phospholipids in line with MD simulation’s observation. This non-specific interaction may arise only at high surface concentration as a result of steric volume effect in solution.

Together, fluorescence based cell viability, membrane potential neutralization, electron microscopy, dipole potential and fluorescence anisotropy data suggest the concentration dependent membrane deformation by nisin in both *B. subtilis* and *E. coli* validating our hypothesis derived from MD simulations. We thus propose that the resulting striking reduction in the surface area/size as a result of membrane deformation is not only due to the inhibition of lipid II mediated cell wall synthesis as previously established^8^ but also a consequence of significant loss of other membrane lipids.

## Discussion

In this study, we investigate the direct role of nisin-phoshpholipid interaction in driving lipid II-independent contribution to the membrane deformation apart from established lipid II-dependent mechanism. Using MD simulations and experimental approaches we show that nisin can deform the membrane through non-specific interaction with phospholipids upon crowding on the membrane surface of both gram-positive (lipid II rich) and gram-negative bacteria (lipid II deprived). We further validate this at molecular level by reconstitution approach showing significant interaction of nisin with phospholipids in a concentration dependent manner.

We observed from the MD simulation analysis that insertion of nisin lead to the destabilization of the native membrane curvature, symmetry, lipid-packing density for both single/mixed lipid systems and also resulting in the formation of water cavities referred to as “water defects”, as similar effects on membranes were also observed for by other antimicrobial peptides as reported earlier^22^,^23^,^24^. The MD data from the last frames of simulation trajectories showed that in the absence of nisin, there was no water translocation appearing in the POPC bilayers. But the addition of monomer and oligomers of nisin peptide leads to entry of water molecules in the membrane. The strongest water permeation was found in case of pentameric nisin insertion resembling efflux channel like forms reported earlier^25^. Furthermore, density analysis of individual head-groups and tail-groups suggested membrane thinning in the Z-dimension disrupting and lowering the overall lipid packing density in the membrane (Supplementary Fig. S1). The increase of POPS head-group density in the box centre showed that POPS molecules were interacting more with the nisin peptide than the other lipid molecules and this observation was again validated by the distance calculation analysis. This also supports our experimental data showing strong interaction of nisin with DOPS membrane as confirmed by dipole potential, fluorescence anisotropy, intensity, and membrane surface potential changes (Fig. 4). The insertion of nisin was found to cause expansion of lipid bilayer in the XY plane and membrane thinning along the Z-dimension, lowering the overall lipid packing density in the membrane and acyl chain order (Supplementary Fig. S1). Increment in the motional anisotropy of the hydrophobic tails results in membrane expansion in the XY plane and thinning in the Z dimensions^26^,^27^. The degree of disorder was high near the head region for the sn2 chain due to its closer positioning to the head-group^28^. On the contrary, lower degrees of freedom in the movements restricted by the rigid nature of the double bond led to low disorderliness in the sn1 chain of the POPC lipid molecules^29^. POPS lipids were found to have highest disorder in the deuterium order parameters for both sn1 and sn2 in comparison to the POPC and POPE lipid molecules as also reported earlier^26^,^30^. We, thus, hypothesized that the degree of oligomerization of the peptide and membrane deformation is dependent on the peptide crowding in the bacterial membrane that in turn is dependent on the concentration regime of the peptide. More deviation in the order parameter of POPS acyl chains may be due to more interactions between oppositely charged POPS and nisin molecule which resulted in more motional anisotropy in the case of POPS acyl chains as compare to the POPC acyl chains. The similar observation is visible from decreased dipole potential of DOPS membranes in the presence of nisin.

To experimentally validate our hypothesis, we exposed *B. subtilis* (lipid II rich) and *E. coli* (lipid II deprived) with nisin and found significant prolongation of the lag phase with increasing concentrations in a range of 0.4 to 12 μM (Fig. 2a–b). The rationale for selecting this particular range was drawn from the growth kinetics showing ineffectiveness of 0.4 μM (or 50 μg/mL) nisin in inducing lag or predominant cell death reported earlier^15^,^31^. Surprisingly, the bacterial population in the extended lag-phase were found to recover and progress through exponential phase suggesting that extended lag time could be a tolerance adaptation by bacterial cells against antimicrobial treatment pressure^32^,^33^. Previously, it was observed that 1 to 100 nM of nisin induced rapid cell death in gram-positive *M. flavus* established based on an 8-hour growth kinetics^8^,^34^. However, we observed that the bacterial cell population was found to recover after extended-lag in both *B. subtilis* and *E. coli* (Fig. 2a–b). While the response of *M. flavus* and *B. subtilis* to nisin might not be comparable, however, considering the similar doubling times and experimental conditions, it appears that the time scales of the growth kinetics is critical in capturing the heterogeneity in the cell viability of the diverse bacterial population in real time (Supplementary Fig. S6). On the contrary, in another study nisin (i.e, concentration regime of 5–50 μg/mL or 0.04–0.4 μM) induced membrane permeabilisation of *B. subtilis* led to accelerated cell division and resulted in late cell death due to inhibition of cell-wall synthesis without showing any immediate effects^15^. However, higher concentrations of nisin treated during the mid-exponential phase do result in immediate drop in absorbance as can be seen from growth curves (Supplementary Fig. S4). Further, we also found that the bacterial populations treated with nisin reaching the stationary phase were able to rescue themselves, although not completely, after inoculating them in fresh culture devoid of any nisin (Supplementary Fig. S5). Together, the growth kinetics that we report suggest that time scale of growth kinetics and the stoichiometry of the peptide (as inferred from both experiments and simulations), could lead to different observations and thus, asks for more caution in MIC based conclusions. Strikingly similar effects of nisin on *E. coli*, that has far lesser lipid II molecules compared to gram-positive bacteria^21^, also hints at a more generalized mechanism of bacterial cell death that may be initiated through the established lipid II interaction but driven by alternative mechanisms causing membrane deformation. This is clearly evident from the fluorescence-based assay that suggests more than ~90% inhibition of cell viability for the population of bacteria present in the stationary phase as a result of nisin treatment at high concentration (12 μM), both in case of *B. subtilis* and *E. coli* (Fig. 2c–h).

Electron microscopy observations of the changes in morphology of *B. subtilis* and *E. coli* as a result of treatment of low and high concentrations of nisin further supported our hypothesis (Fig. 3). Exposure to 0.4 μM of nisin did trigger minor changes in the size; however, the shape at large remained similar in case of *B. subtilis* (Fig. 3b). However, exposure to 12 μM of nisin resulted in a five-fold reduction in the surface area of *B. subtilis* (Fig. 3e and r) and significant bleb-like membrane protrusions in *E. coli* (Fig. 3g). To address the observed recovery in growth kinetics, we further captured the changes in membrane morphology during various growth phases. The changes in morphology suggest early recovery during transition from late lag to mid-exponential phase as evident by the appearance of septating cells. This could be due to accelerated cell division resulting in maximum reduction in surface area during transition from exponential to stationary phase^13^,^15^,^35^. The dissipation of membrane potential, that is otherwise essential for normal cell division and growth, could be responsible for the accelerated cell division as a result of delocalization of morphogenic and cell division proteins followed by cell death^36^. The subsequent drastic reduction in surface area could be due to a higher peptide density on the membrane surface of septating bacterial cells as a result of lower surface area spread along the chain in line with similar observation made with other peptides such as LL-37^37^. This is evident from the correlation between nisin concentrations, membrane potential neutralization and cell viability (Fig. 2n and o) suggesting the role of peptide crowding in causing cell death^38^.

To decipher the lipid-II independent interaction with phospholipids at the molecular levels we picked DOPS owing to its negative charge. The decrease in the dipole potential of DOPS membranes revealed perturbations in the non-random arrangement of molecular dipoles in membrane interface by higher concentration of nisin (Fig. 4b). Likewise, the decrease in relative intensity of di-8-ANEPPS in DOPS membranes can be attributed to increase in the polar environment around the fluorophore, which reduces its fluorescence lifetime by providing an alternative, non-radiative path back to the ground state (Fig. 4d). Further, uniform decrease in fluorescence polarization and anisotropy of DOPS membranes revealed decreased rotational relaxation time, a function of membrane viscosity (Fig. 4f). The orientation of transition dipole of dye is changed during the lifetime of excited state due to increased mobility. Such fluidizing effect of nisin is not observed in case of DOPC membranes where motional restriction on the fluorophore is not relieved. Such quantitative differential effects in membrane surface and dipole potential, fluorescence intensity and anisotropy particularly in DOPS membranes with increasing concentration of nisin suggesting the existence of lipid-II independent non-specific interaction in line with previous reports^39^,^40^,^41^. This could also explain the unspecific rupture of negatively charged DOPG membranes by micromolar concentration of nisin which were prevented by lipid-II^42^.

It is estimated that there are about 10^5^ bactoprenyl-phosphate pool available for lipid II synthesis in gram-positive bacteria and about 2000 lipid II molecules present in the cell membranes of gram-negative *E. coli*^43^, which is far less in number as compared to other phospholipids (see Supplementary Table S3 *for an approximate estimation of number of lipids*). As the lipid II mediated pores formed by nisin are reported to be stable only for few seconds^12^ because of the opposing line tension imposing closure of the pore^44^. We reasoned that while nisin’s specific interaction with lipid II initiates membrane deformation^45^, the interaction of non-lipid II binding region of nisin with the phoshpholipids could be critical in driving the deformation stronger. The truncated C terminus of nisin has been found to cause 100-fold reduction in the bactericidal effect of nisin^46^ suggesting the role of the non-lipid II binding region of the peptide in pore formation and possible non-specific interactions with surrounding lipid molecules as proven by our anisotropy and dipole potential data.

**Figure 5:**
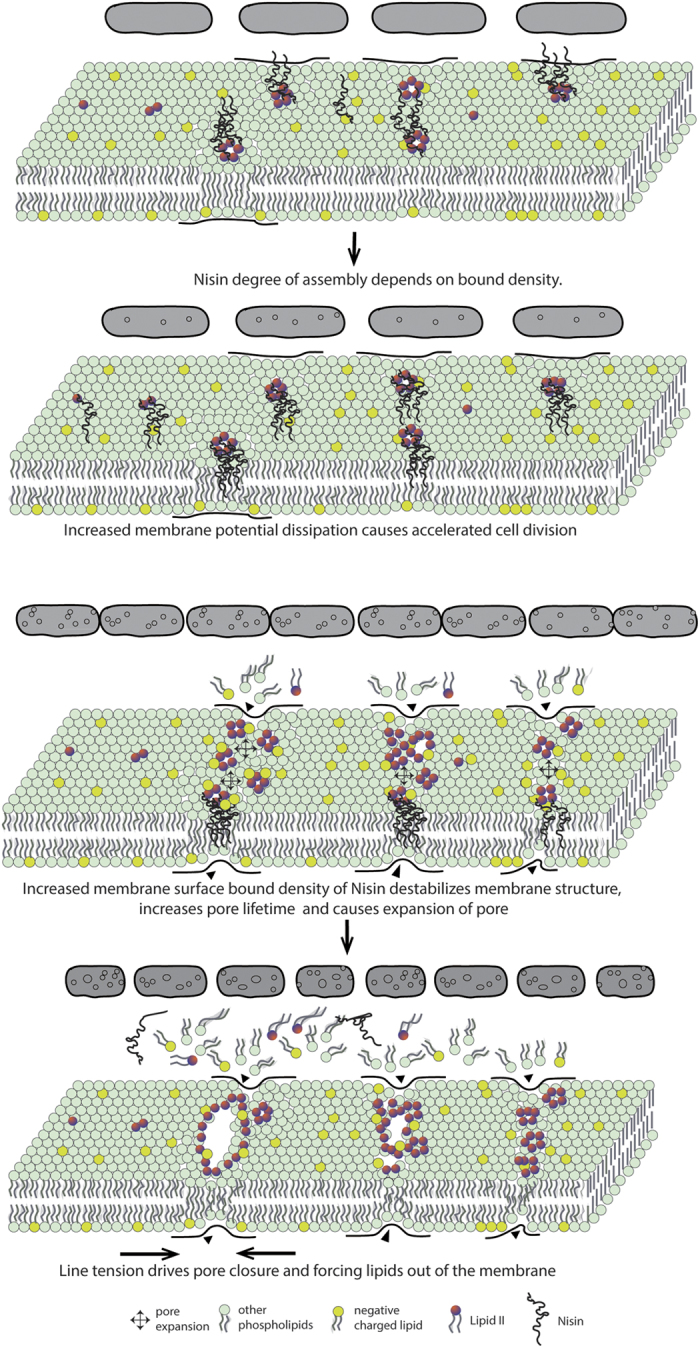
Generalized physical model depicting the interaction of nisin with bacterial cell membrane particularly focusing on the relationship between peptide’s bound density and membrane deformation.

We propose a general physico-chemical mechanism wherein the degree of peptide crowding and oligomerization may drive lipid II independent membrane deformation. This is particularly evident from the observations on *E. coli*, whose viability is equally affected despite having far lesser lipid II in its membrane as observed from failure to recover completely after extended lag phase in growth kinetics (Fig. 2b) as well as LIVE/DEAD backlight cell viability assay (Fig. 2i). High concentration of nisin was found to trigger significant membrane deformation (i.e, membrane protrusions, pores and bleb-like structures) (Fig. 3c,d,g,h), particularly, during transition from mid-log to stationary phase (Fig. 3k,l,o,p) as shown from changes in morphological feature of both of the bacteria. Peptide crowding, as a result of high concentration, eventually causes significant membrane distortions (like pore formation) inducing changes in membrane anisotropy, dipole potential (Fig. 4) as well as lipid order and packing density (Fig. 1). The driving force for pore formation seems to be the membrane tension generated because of the peptide density on the surface of bacterial membrane in line with other reports^47^. Line tension driven merging and expansion of the pores thereby stabilizing the pore lifetime^44^,^47^ strongly supporting our observations. We believe that the morphological changes occurring in a collective bacterial population upon exposure to nisin depend on the amount of peptide crowding on the surface. At low surface bound density, the weak oligomerization of the peptide would trigger membrane distortions that are recovered. At moderate surface bound density, the peptide may induce accelerated cell division and even cell death to some extent, however, followed by recovery through tolerant mechanisms in a collective population. On the contrary, high surface bound density facilitates strongest oligomerization causing stabilization of pores, membrane potential dissipation and triggering excessive loss of lipids from the bacteria membrane (Fig. 5).

Such protein-protein crowding causing extensive membrane deformation has also been reported for various other proteins^48^,^49^. Existence of diverse populations of bacteria in culture with different amount of surface bound peptide may represent variable cell death observed in the past^8^,^15^. Together, our results highlight the importance of the membrane surface stoichiometry of the antimicrobial peptide and the time scale of growth kinetics in capturing the heterogeneity of collective population of bacteria. It would be interesting to explore further the potential mechanism through which lipid homeostasis is affected by the excessive loss of phospholipids of membrane leading to five-fold reduction in the surface area. The present study will help in better understanding the mechanism of not only nisin but also other antimicrobial therapeutic peptides, and further pave the way towards their applications in clinical settings.

## Materials and Methods

### Materials

Nisin from Sigma Aldrich (USA). Sodium phosphate monobasic and sodium phosphate dibasic salts were purchased from Sigma Aldrich (USA). Glutaraldehyde (25%) and Tannic acid used for electron microscopy study were purchased from Merck (India). Nutrient broth, nutrient agar, Mueller Hington broth, HEPES, sodium chloride and ethanol, methanol, chloroform were bought from HiMedia, India. Live/Dead BacLight Bacterial viability Kit for Live/Dead assay was purchased from Molecular Probes, Invitrogen, India. The strains, *Bacillus subtilis* (MTCC 736) and *E. coli* (MTCC 443), used for antimicrobial study were purchased from Institute of Microbial Technology (IMTECH), Chandigarh, India. Lipids were purchased from Avanti polar lipids, USA.

## Methodology

### Parameters for Molecular Dynamics simulation of nisin peptides embedded in the lipid bilayer

1-palmitoyl-2-oleoyl-sn-glycero-3-phosphocholine (POPC) pure and POPC, 1-palmitoyl-2-oleoyl-sn-glycero-3-phosphoethanolamine (POPE) and 1-palmitoyl-2-oleoyl-sn-glycero-3-phospho-L-serine (POPS) mixed bilayers were used for the MD studies. POPC bilayer with 128 lipid molecules and 2460 water molecules co-ordinates of model membrane were derived from Peter Tielman’s website (wcm.ucalgary.ca/tieleman/). The mixed bilayer consisting of 102 POPC, 14 POPE and 12 POPS lipid molecules was reconstituted using MemGen^50^ (memgen.uni-goettingen.de) webtool. The bilayers were energy minimized first and equilibrated at 310 K temperature (NVT) and 1 atm pressure (NPT) conditions. 40 ns of Molecular Dynamics (MD) simulation sessions were then performed for the POPC and POPC + POPE + POPS (PC/PE/PS) bilayer to analyze the local membrane properties in the absence of peptide molecule. The PDB file of nisin antimicrobial peptide was derived from Protein Data Bank (PDB ID: 1WCO). As this PDB file is a complex of nisin with lipid–II chains, so the protein chain was separated from the complex and remodeled using Phyre2^51^. The region of the protein from 6–34 residues was observed in the model. This region was containing non lipid-II interacting amino acids. In order to study the effect of interaction of monomer and oligomeric form of nisin with POPC membrane, oligomers of nisin were generated using symmdock^52^ program as explained earlier. Tetrameric and pentameric forms of nisin were selected due to their symmetrical channel like structures for analyzing the effect of their insertion on the properties of lipid membrane. The AMP-Lipid bilayer systems were generated in which the efficient insertion of peptide molecules in the lipid bilayer was carried out using LAMBADA tool and these systems were optimized using Inflategro script^53^.

### Membrane destabilization measurements

This kind of analysis using MD simulations to study the properties of biological membrane remains an efficient way^54^,^55^. It may provide an overview of lipid molecules that interact with the peptides embedded in the lipid bilayer and affect the activity of the lipid bilayer and *vice-versa*^56^. The results of simulations of lipid membrane i.e. area per lipid, root mean square deviation (RMSD), deuterium order parameter were compared with the standard observations from X-ray and NMR experiments to observe effect of protein interaction on lipid acyl chains in the model membranes^57^. Gromacs package^58^ was used to carry out simulation of pure POPC, PC/PE/PS bilayers and all peptide-lipid complexes. In this study, the Gromos 53a6 force field was used for simulations in all the systems. To provide natural physiological cellular environment, the temperature equilibrations were carried out at 300 K and 310 K respectively, while the pressure equilibrations were carried out at 1 bar atmospheric pressure. Different parameters like deuterium order parameter of acyl chain, density of the phosphate head-groups and acyl chains of lipid molecules, root mean square deviations (RMSDs) of atomic positions and water defects were calculated using different utilities of Gromacs package 5.0 along the trajectory during the simulations from 10 ns to 40 ns. We plotted the order parameter *versus* position of carbon atoms. The carbon positions have been renumbered from 2 as the order parameter calculation using g_order yields the order parameters from C_2_ to C_n−1_ positions only, where n signifies the number of C atoms in the chain. The density of head-groups and tail-groups were computed along the Z-dimension of the simulation box for pure POPC bilayer as well as for all the peptide-lipid complexes. The area per lipid and membrane curvature was computed using g_lomepro_v1.0.2^59^ that were later analyzed using XMGRACE^60^, Gnuplot 5.0 and VMD^61^ respectively.

### Preparation of nisin solution

Nisin stock was prepared in 10 mM HEPES (pH–7.4) containing 150 mM NaCl and subsequently filtered through 0.22 μm syringe filter prior to use^62^,^63^. For fluorescence measurements nisin stock was prepared in 280 mM sucrose supplemented with 5 mM Tris (pH 7.5) buffer. For all working concentrations of nisin further dilutions were made using the same buffer.

### Growth kinetics

MIC is the minimum concentration of antimicrobial agent that inhibits the growth of bacterial population upto 50%. Microplate dilution method was used to determine the MIC of nisin in nutrient broth (NB). For experimental work, the mother cultures of *B. subtilis* and *E. coli* were prepared by inoculating a loop full of bacteria from slant cultures in NB and incubated at 120 rpm, 37 °C, till OD_600nm_ (Optical Density) of 1.0. 20 μl mother cultures were mixed with increasing nisin concentrations (0.4, 1.5, 4, 8, and 12 μM) in a 96 well microplate, and total volume in each well was adjusted to a final volume of 300 μL using nutrient broth. For control, reaction mixtures were made from mother cultures in nutrient broth without nisin. The growth kinetic studies were performed by measuring optical density at regular time intervals at 37 °C with constant shaking for 20 hours using plate reader (Synergy H1 hybride reader, Biotek, USA). Treatments were done in triplicate for control and each sample treated with nisin to calculate mean and standard error. The assay was performed thrice on different days.

### Minimum Bactericidal Concentration (MBC)

MBC is defined as the minimum concentration of antibiotic required for reducing the viability of bacterial population upto 99.9%. 10 μl bacterial samples treated with different concentrations of nisin were collected from the stationary phase of growth kinetics and diluted to 10000 times using autoclaved distilled water. From these diluted stocks 10 μl was spread on nutrient agar plates, and incubated overnight at 37 °C followed by counting the colonies to determine the MBC. The colony-forming unit CFU/mL was calculated using the following formula.


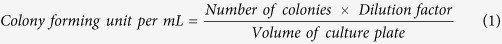


### Membrane surface potential dissipation analysis

Surface charge measurement of *B. subtilis and E. coli* as a result of treatment of nisin was performed by Zeta potential investigations. For the experiment, mother cultures of bacterial strains were done in Mueller Hington broth (MHB) taking loop full of bacteria from slant cultures, followed by overnight incubation at 37 °C and 150 rpm. 300 μl of bacterial cultures were then transferred to freshly prepared 5 mL MHB, and incubated at 37 °C to obtain an OD_600nm_ ~0.3. The cultures were then centrifuged at 13000 rpm for 8 minutes, and the obtained pellet were washed twice with 10 mM HEPES buffer (pH-7.4) containing 150 mM NaCl. The pellets were then resuspended in HEPES buffer. Different concentrations (i.e, 0.4, 1.5, 4, 8 and 12 μM) of nisin were prepared by dissolving required amount of nisin in 10 mM HEPES (pH-7.4) having 150 mM NaCl. For the surface interactions study, 100 μl of prepared nisin solution was added to 900 μl of bacterial suspension, and incubated for 2 hours at room temperature. Control samples had only bacterial culture suspended in HEPES buffer without nisin. To study the effect of nisin on individual lipids, measurement of surface charge potential of large unilamellar vesicles (LUVs) made from individual lipids was performed. For LUV preparation, 400 nmoles of each lipid from stock solution was taken. The lipids were dried in a vial with nitrogen stream and placed in vacuum for 2 hours for evaporation of remaining residual solvent. 1 mL of PBS buffer (pH-7.4) was added, and the temperature of the solution was raised above the melting temperature of lipids, vortexed and sonicated. The sonication was set at a frequency of 80 Hz for 0.8 seconds cycle. To measure zeta potential and the size of LUVs, the above solution was 5 fold diluted with buffer. Nisin was then added to LUVs in 9:1 ratio with increasing concentrations from 0.4, 1.5, 4, 8 and 12 μM. For control only 1 mL of LUV in buffer was taken. These solutions were incubated for 2 hours to determine the surface charge potential on lipids.

The Zeta potential studies were done on a Zetasizer Nano ZS (Malvern Zetasizer Nano ZS90, Netherland) set with a 633 nm Laser. Treated bacterial samples and LUVs of each lipid type were transferred to cuvette having gold electrodes and allowed to equilibrate for some time at 25 °C. After equilibration, surface potential of each sample was calculated from Smoluchowsky equation by determining electrophoretic mobility. This experiment was done in triplicates for each concentration of nisin using fresh cultures as mentioned above.

### Fluorescence based bacterial cell viability assay

Bacterial cell viability was carried out using LIVE/DEAD BacLight viability kit (L007, Molecular Probes, Invitrogen). For this assay the protocol suggested by manufacturer was followed and imaging was done by Fluorescence microscope (Olympus, IX71) with an objective of magnification 20X. The controls for this assay were bacteria without treatment of nisin and the tests were bacteria treated with 12 μM of nisin to visualize viable and non-viable bacterial cells. The assay was done thrice for calculation of percentage of bacteria killed after introduction of high dose of nisin.

### Electron microscopy of bacterial surface morphology

For insight into the surface morphology of *B. subtilis* and *E. coli* upon treatment with nisin, the bacterial samples were visualized under FE-SEM. For sample preparation, bacteria were collected in early stationary phase from 96 well-plate, incubated for growth kinetics treated with 0.4 and 12 μM of nisin. 1 mL of each bacterial sample was centrifuged at 5000 rpm for 5 minutes at 4 °C then pellet obtained was washed with 1 X PBS buffer (pH-7.4) twice. After washing pellet was resuspended in PBS and one drop of resuspended culture was spread on glass slide, fixation was done by incubating slides in 2.5% glutaraldehyde. The slides were washed with 1% tannic acid for few minutes, then again washed with distilled water. Sequential dehydration was done with 30%, 50%, 70%, 90%, and absolute ethanol particularly in that order. These dehydrated samples were coated by gold and platinum to visualize under SEM (Jeol - JSM 6480-LV SEM, Japan) and FE- SEM (Nova NanoSEM 450/FEI) respectively.

### Reconstitution experiments for dipole potential and fluorescence anisotropy

For each sample, 120 nmol of corresponding lipid (in chloroform) and 1.2 nmol of di-8-ANEPPS (in methanol) aliquots were mixed to achieve a dye:lipid molar ratio of 1:100 (dye was not added in background samples). Samples were dried under stream of nitrogen gas and then incubated in vacuum chamber for at least 2 hours to ensure complete evaporation of organic solvent molecules. Thereafter lipids were suspended in 500 μL of 280 mM sucrose supplemented with 5 mM Tris (pH 7.5) buffer. After hydration, the suspension was heated in water bath for 15 minutes at 45 °C, vortexed for 3 minutes and then sonicated for 5 minutes to produce LUVs of roughly (200 nm) diameter which was confirmed by dynamic light scattering. 100 μL of increasing concentrations of nisin solutions were added to the liposomes giving rise to a final volume of 600 μL per sample with 200 μM lipid, 2 μM dye and 0, 5, 10, 15, 20, 25 μM nisin. Treated samples were incubated in dark for two hours at room temperature (~24 °C) before measurement. All measurements were performed by LS55 PerkinElmer spectrofluorimeter with 500 μl quartz cuvette. Excitation and emission slits with bandpass of 5 nm were used throughout. The excitation spectra were obtained at emission wavelength of 670 nm to avoid membrane fluidity effects^64^ using a scan speed of 240 nm/s. Corresponding background intensities were subtracted from all test samples containing di-8-ANEPPS. The ratio (R), defined as the ratio of emission intensities at 670 nm due to excitation at 420 nm and 520 nm, was calculated from the excitation spectrum of each sample and plotted as a function of increasing concentration of nisin. Fluorescence intensity and anisotropy were monitored for 10 s and 20 s, respectively, with integration time of 1 s. The excitation and emission wavelengths were set to 460 nm and 560 nm, respectively. All anisotropy (r) values were automatically calculated by the instrument from the equation^65^.


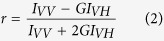


where, I_VV_ and I_VH_ are the measured fluorescence intensities with excitation polarizer oriented vertically and emission polarizer oriented vertically and horizontally, respectively. G (I_HV_/I_HH_) is the grating correction factor that corrects for wavelength-dependent distortion of the polarizers. All experiments were conducted with multiple sets of samples.

### Calculation of bacterial dimensions

The number of pixels on the scale bar was taken as reference for the calculation of dimensions (i.e. length, breadth and surface area) of the bacteria using Image J. The surface area was determined by assuming the bacteria to be approximately a cylinder using the given equation


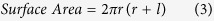


where, r is the half of the breadth and l is length of a bacterial cell.

No conclusion could be drawn from *E. coli* micrographs due to excessive deformation of the membrane surface.

### Image Processing

All the images were processed and analyzed with Image J. Surface roughness analysis was measured using the surface roughness profile option in Image J after background correction.

### Statistical analysis

Statistical analysis were carried out using Origin Lab and Stata data analysis and statistical software. Paired student’s t-tests were performed to evaluate differences in length and surface area of *B. subtilis* without and with varying concentration of nisin treatment. Changes in the dimensions of bacteria under different conditions (i.e, control, nisin-treated) were calculated for 50–60 bacteria from three different sets of experiments. One-way analysis of variance was carried out to compare the control and different conditions of nisin-treatment of bacteria. P values ≤ 0.05 were considered to be significant for all the analysis, which were indicated by asterisks: ***P ≤ 0.001.

## Additional Information

**How to cite this article**: Prince, A. *et al*. Lipid-II Independent Antimicrobial Mechanism of Nisin Depends On Its Crowding And Degree Of Oligomerization. *Sci. Rep.* **6**, 37908; doi: 10.1038/srep37908 (2016).

**Publisher's note:** Springer Nature remains neutral with regard to jurisdictional claims in published maps and institutional affiliations.

## Supplementary Material

Supplementary Information
