# Corrigendum: Lipid-II Independent Antimicrobial Mechanism of Nisin Depends On Its Crowding And Degree Of Oligomerization

**DOI:** 10.1038/srep41346

**Published:** 2017-02-10

**Authors:** Ashutosh Prince, Padmani Sandhu, Pankaj Ror, Eva Dash, Shingarika Sharma, Manoranjan Arakha, Suman Jha, Yusuf Akhter, Mohammed Saleem

Scientific Reports 6: Article number: 37908; 10.1038/srep37908 published online: 11 29 2016; updated: 02 10 2017.

The original version of this Article contained an error in the spelling of the author Pankaj Ror which was incorrectly given as Pankaj Kumar. This has now been corrected in the PDF and HTML versions of the Article.

