# Supplementary material for: Lipid-II Independent Antimicrobial Mechanism of Nisin Depends On Its Crowding And Degree Of Oligomerization
Source: Sci Rep. 2016 Nov 29;6:37908. doi: 10.1038/srep37908 (PMC5126574; doi:10.1038/srep37908)
Supplement: Supplementary Information [file srep37908-s1.docx]

**Supplementary Information**

**Full Title:**

**Lipid-II Independent Antimicrobial Mechanism of Nisin Depends On Its Crowding And Degree Of Oligomerization**

Ashutosh Prince^1^, Padmani Sandhu^2^, Pankaj Kumar^1^, Eva Dash^1^, Shingarika Sharma^2^, Manoranjan Arakha^1^, Suman Jha^1^, Yusuf Akhter^2,3^, Mohammed Saleem^1,3^*

^1^ Department of Life Sciences, National Institute of Technology, Rourkela, India.

^2^ Centre for Computational Biology and Structural Bioinformatics, School of Life Sciences, Central University of Himachal Pradesh, India.

^3^ Co-senior authors

*Corresponding author: saleemm@nitrkl.ac.in

**Supplementary Text**

**Nisin induces changes in membrane curvature and thickness**

Lipid bilayer possesses curvature due to hydrophobic and hydrophilic interactions between the lipid molecules as well as their interactions with the polar environment (Supplementary Fig. S1a). After the amphipathic helix of nisin was inserted in the membrane, it was observed to interact with the neighboring lipids and pulled them closer causing a shift in the center of curvature towards the peptide. (Supplementary Fig.S1b) shows that insertion of nisin monomeric peptide in lipid membrane generated local curvature at the point of insertion of the nisin in the bilayer due to various interactions. Upon insertion of tetrameric and pentameric oligomers of nisin, small curvatures were found to spread over a large surface area. These higher oligomers acquired a channel like conformation and each peptide was observed to be interacting with the lipid molecules and pulling them closer and thereby disturbing the normal bilayer symmetry (Supplementary Fig. S1c and S1d).

**Oligomerization dependent destabilization of lipid bilayer**

Root Mean Square Deviation (RMSD) and the areas per lipid (APL) analysis were performed for all peptide-lipid complexes. Increase in the RMSD was observed in general with higher degree of nisin oligomerization (Supplementary Fig. S2a and S2b). The average APL of the POPC bilayer in the presence of the nisin monomer and two different oligomers are shown in Figure S2c. It can be visualized from the plots that APL decreased from an initial value of 0.68 nm^2^ to the value of 0.60 nm^2^ and stabilized with little fluctuation of 0.1 nm^2^ in the case of peptide-lipid complexes with POPC bilayer. For PC/PE/PS bilayer the APL varied between 0.64 nm^2^ and 0.58 nm^2^ (Supplementary Fig. S2d). An initial continuous decrease in the average APL values were observed to be fluctuating during rest of the simulation time indicating that the insertion of nisin in the lipid bilayer lead to disturbance in the lipid density and consequently in APL.

It was also observed from the results that insertion of the peptide lead to membrane expansion in XY-plane and further caused the thinning of membrane in Z-dimensions. As evident from the (Supplementary Fig. S1a) that membrane is having large distance between upper and lower leaflets in the absence of the nisin peptide but with the subsequent addition of nisin peptide in monomer and two oligomeric (tetramer and pentamer) forms (Supplementary Fig. S1b and S1c), the distance between upper and lower leaflet decreases consequently indicating the thinning in the Z-direction.

**Nisin challenged bacterial populations in log phase**

We then investigated the effect of nisin on *B. subtilis* and *E. coli* when challenged with nisin in the middle of the exponential growth phase (Supplementary Fig. S4a and S4b). Both the bacterial strains being Ampicillin-resistant did not show any inhibition of growth rate upon mid-exponential treatment with 0.3 mM Ampicillin. Exposure to 0.4 µM nisin triggered only a slight dip in the growth curve that recovered within a span of ~45 minutes. Higher concentrations of nisin (1.5 -12 µM), however, induced a steep fall in the growth curve forcing the bacterial population back into the lag phase ranging from ~2-7 hours in case of *B. subtilis* (Supplementary Fig. S4a) and ~2-16 hours in case of *E. coli* before recovering to exponential growth phase (Supplementary Fig. S4b). In both cases, mid-exponential exposure to nisin resulted in inhibition of growth rate as reflected in the resulting final optical densities of the bacterial population. The resulting recovery of exponential phase in both, *B. subtilis* and *E. coli* after ~7 hours and ~16 hours, respectively, could be attributed to the tolerance developed during the lag-phase by unknown mechanism. We next asked whether the recovered bacterial population that underwent division after extended lag phase simply ceased to grow or died upon reaching the stationary phase.

**Growth recovery kinetics of nisin treated bacteria**

To verify whether the recovered bacterial population resulted in growth inhibited or dead bacteria, we inoculated nisin-treated bacteria that have reached the early stationary phase, into a fresh culture without nisin. We then monitored whether the bacteria could rescue and grow normally. Interestingly, none of the nisin-treated bacterial population from the early stationary phase inoculated into fresh culture media could rescue themselves fully in both *B. subtilis* and *E. coli* (Supplementary Fig. S5a and S5b, respectively). We observed a short lag phase for prior nisin treatment of 0.4 – 8 µM and a lag phase of ~5 hours in case of 12 µM nisin treatment for *B. subtilis*, however, in all cases the final optical density could only reach a value of ~0.6 unlike the ideal growth behavior where the OD tends to reach a value of ~1.2. Similar behavior was observed in case of *E. coli* where the rescued population showed a slightly longer lag phase of about ~ 6-7 hours and reaching an OD of ~0.75. This suggests that nisin induced growth inhibition involves long lasting effect on bacterial population (*B. subtilis* and *E. coli*) that are unable to revive fully despite being grown in fresh culture medium devoid of nisin (Supplementary Fig. S5a and S5b).

**Haemolysis Assay with Nisin**

To investigate the effect of nisin in RBC (Red Blood Corpuscles) of blood plasma haemolysis assay was done. This assay evaluates the haemoglobin release due to RBC lysis when exposed to a drug. For this assay, RBCs were isolated, washed and treated with increasing concentration of nisin i.e. 0, 0.4, 1.5, 4, 8 and 12 µM. Positive control was RBC in lysis buffer.

We observed that all the concentrations of nisin were not able to disrupt RBCs (Supplementary Fig. S11). To confirm it we also recorded absorbance at 595 nm of same samples treated with nisin and positive control. We found there was negligible difference in absorbance of 12 µM nisin treated samples in comparison to negative control whereas there was decrease in absorbance of positive control (table, Supplementary Fig. S11).

**Supplementary Fig. S1**


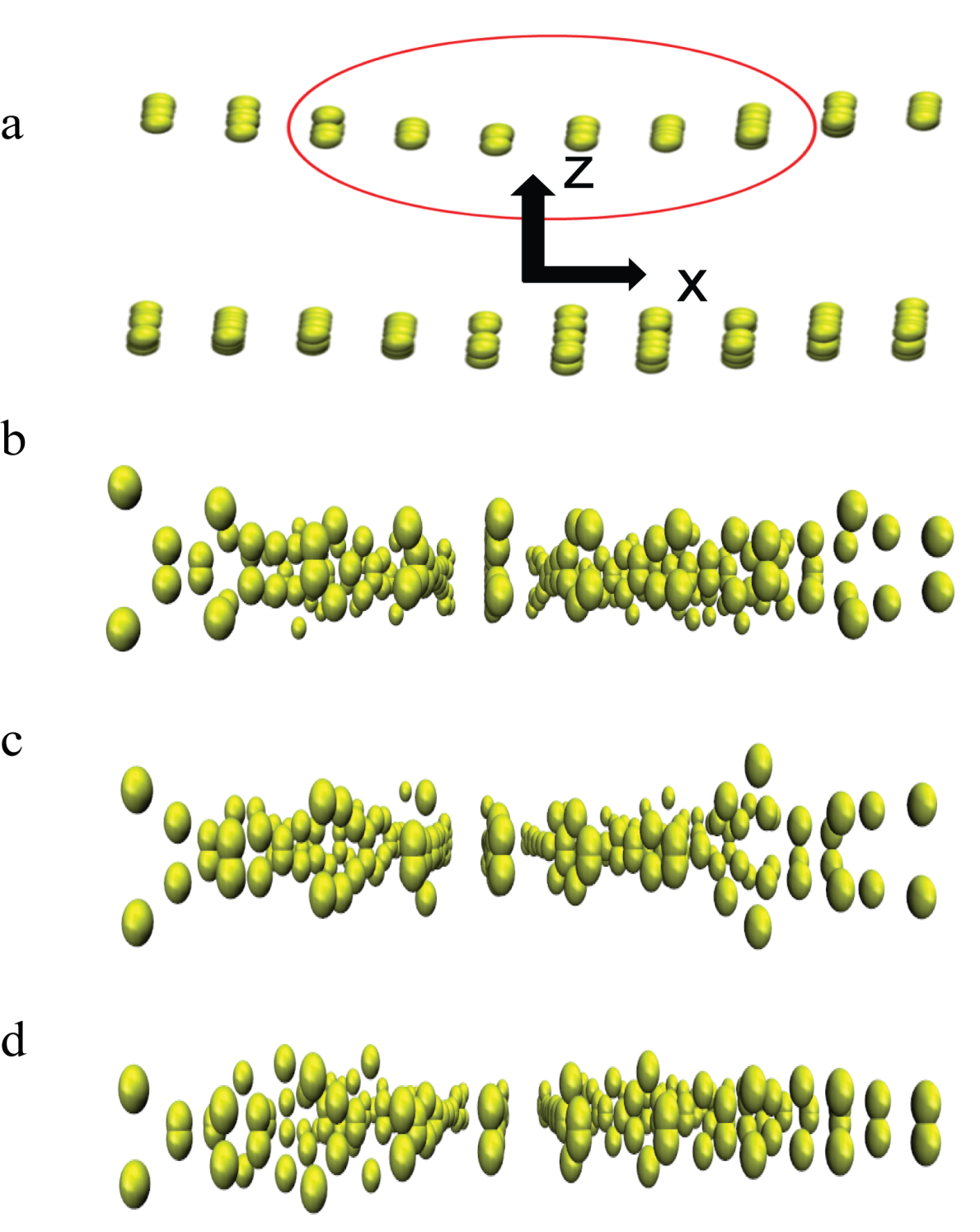


**Supplementary Fig. S1**. **Alteration in lipid packing due to insertion of nisin peptide leads to change in membrane curvatures and permeability.** (a) Pure POPC bilayer with curvature centered in the middle of the membrane. The lipid head-groups are represented in the form of vdw (Van der Walls) model in yellow colour. (b) POPC bilayer with nisin monomer showing smaller curvature and reduced dimensions between the upper and the lower leaflets. (c) Curvature of POPC bilayer after insertion of nisin tetramer expands to a larger surface area around the localization of the peptide. (d) Curvature POPC bilayer with nisin pentamer also shows similar expansions.

**Supplementary Fig. S2**


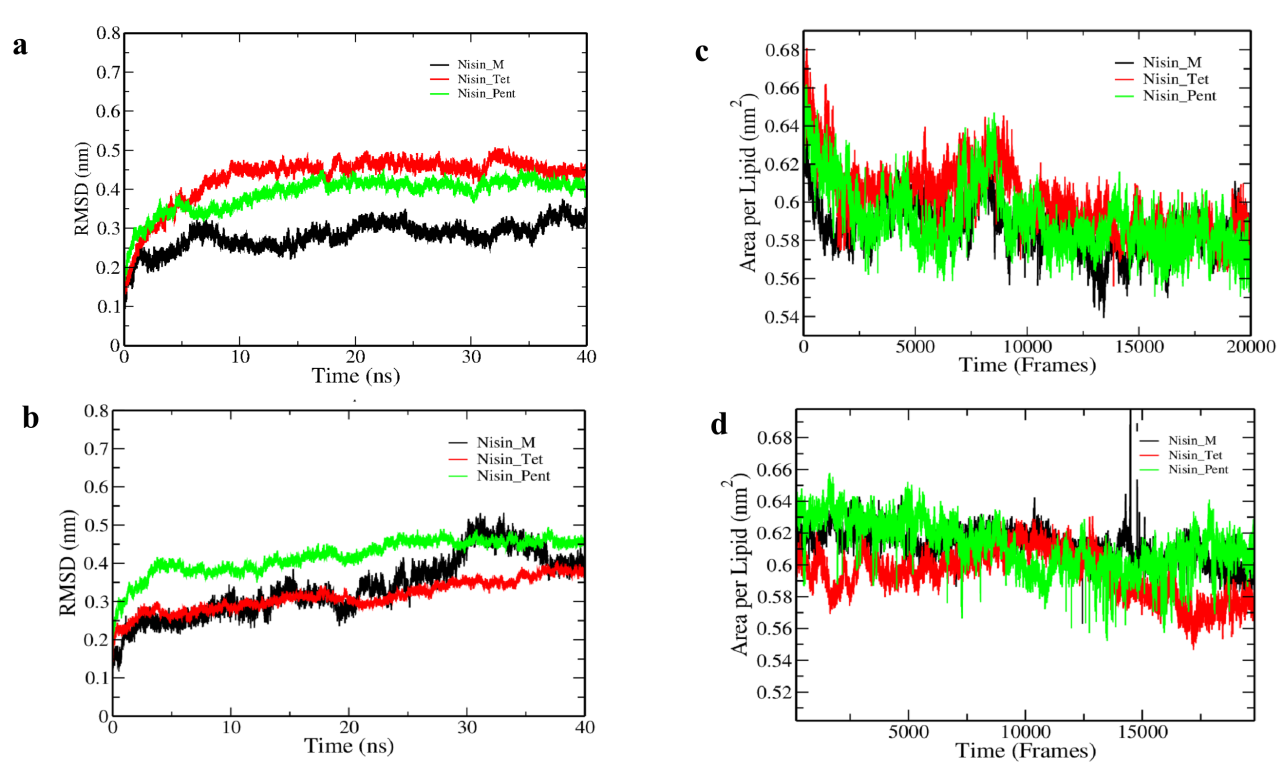


**Supplementary Fig. S2.** **Computation of equilibrated trajectories for MD analysis.** (a) RMSDs of Nisin monomer, tetramer and pentamer in pure POPC bilayer. X–axis represents the time in ns, while the Y-axis represents the RMSDs in nm for each of the complexes. RMSDs of different monomeric and oligomeric form of Nisin were represented in different colour. (b) RMSDs of Nisin monomer, tetramer and pentamer in pure POPC+POPE+POPS bilayer. (c) Average APL of POPC bilayer of different AMP Nisin-bilayer complexes along the simulation trajectories. (d) Average APL of POPC from POPC+POPE+POPS bilayer of different AMP Nisin-bilayer complexes along the simulation trajectories.

**Supplementary Fig. S3**


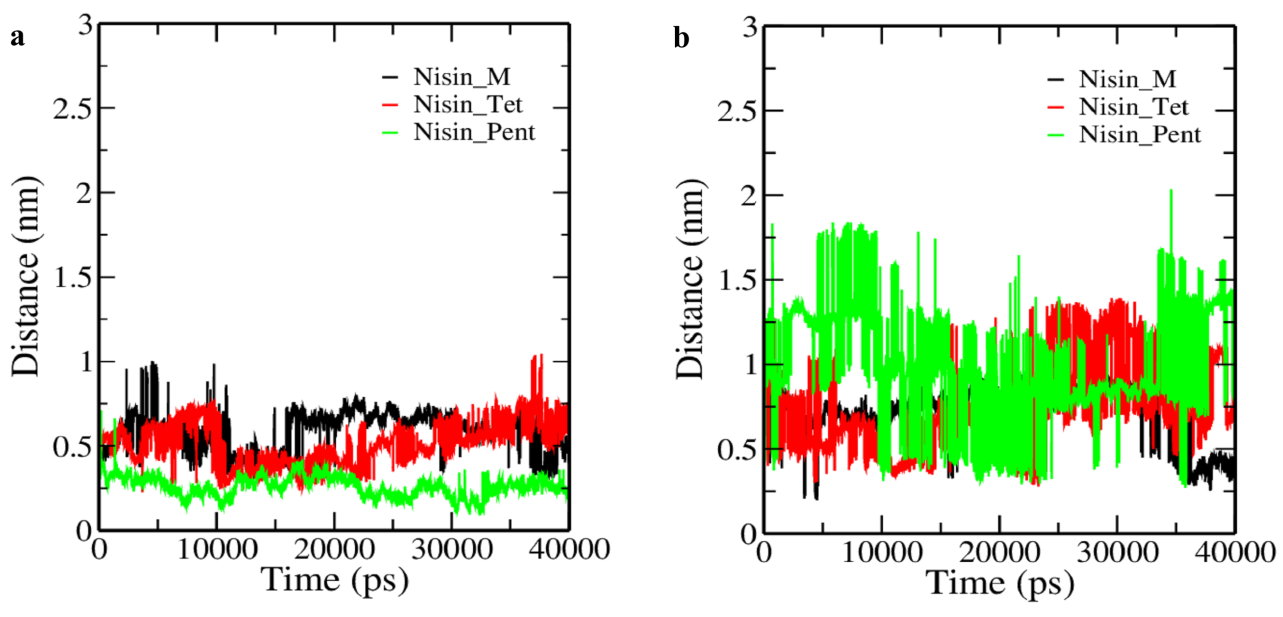


**Supplementary Fig. S3**. **POPS lipid molecules showing comparatively more interactions with Nisin than the other membrane lipids.** X-axis represents the time in ps and Y-axis represents the Distance between the centre of masses of lipid molecules and peptides in nm (a) Distance between the POPE lipid molecules and Nisin antimicrobial peptide monomers and oligomers shown in different colours. (b) Distance between POPS lipid molecules and Nisin antimicrobial peptide monomer and oligomers in different colours.

**Supplementary Fig. S4**


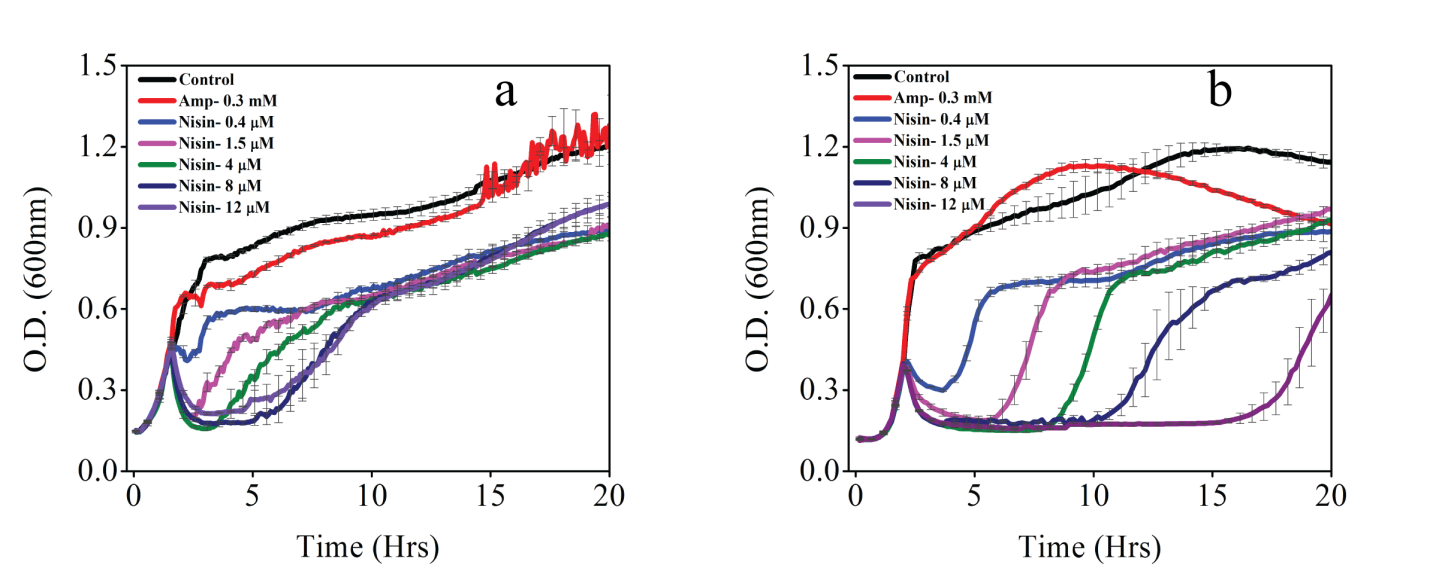


**Supplementary Fig. S4. Effect of increasing concentrations of nisin treatment in mid-exponential phase on the growth kinetics of bacteria (a - *B. subtilis*; b - *E. coli*).**

**Supplementary Fig. S5**


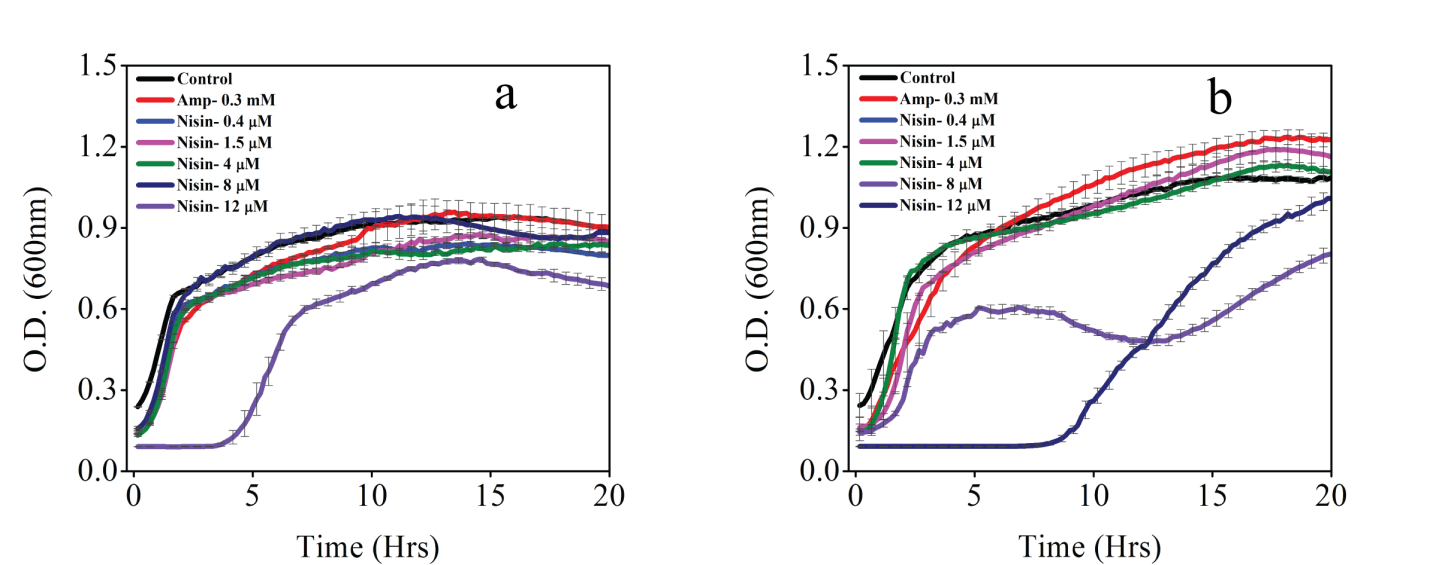


**Supplementary Fig. S5. Effect of increasing concentrations of nisin treatment on the rescue of bacteria from stationary phase inoculated to fresh nutrient media lacking nisin (a - *B. subtilis*; b - *E. coli*).** (Time scale 20 hrs for all experiments, no. of independent experiments, n = 3)

**Supplementary Fig. S6**

**
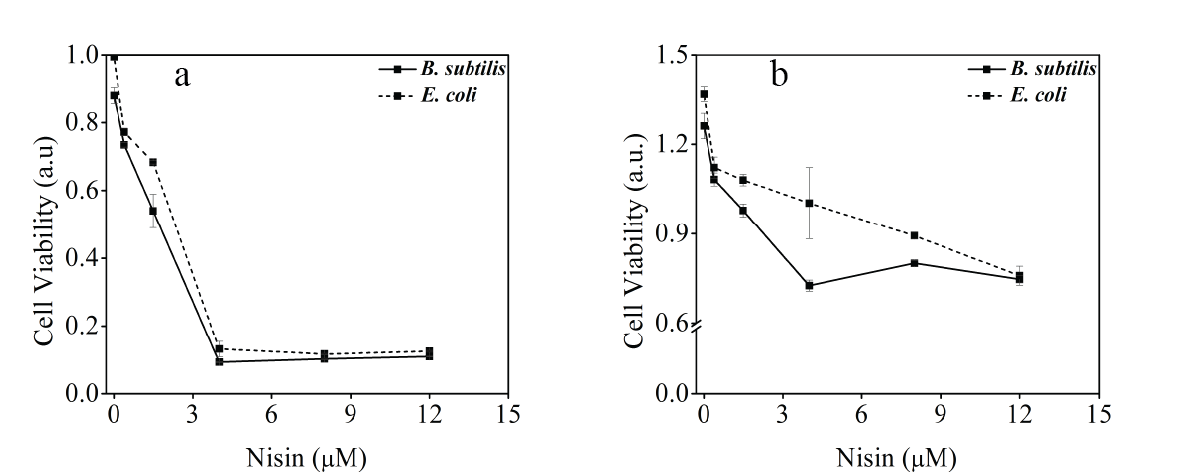
**

**Supplementary Fig. S6. Comparison of the cell viability of *B. subtilis* and *E. coli* considering different time scales of growth kinetics treated with Nisin different concentration**. (a) 8 hour growth kinetics, and (b) 20 hour growth kinetics.

**Supplementary Fig. S7**

**
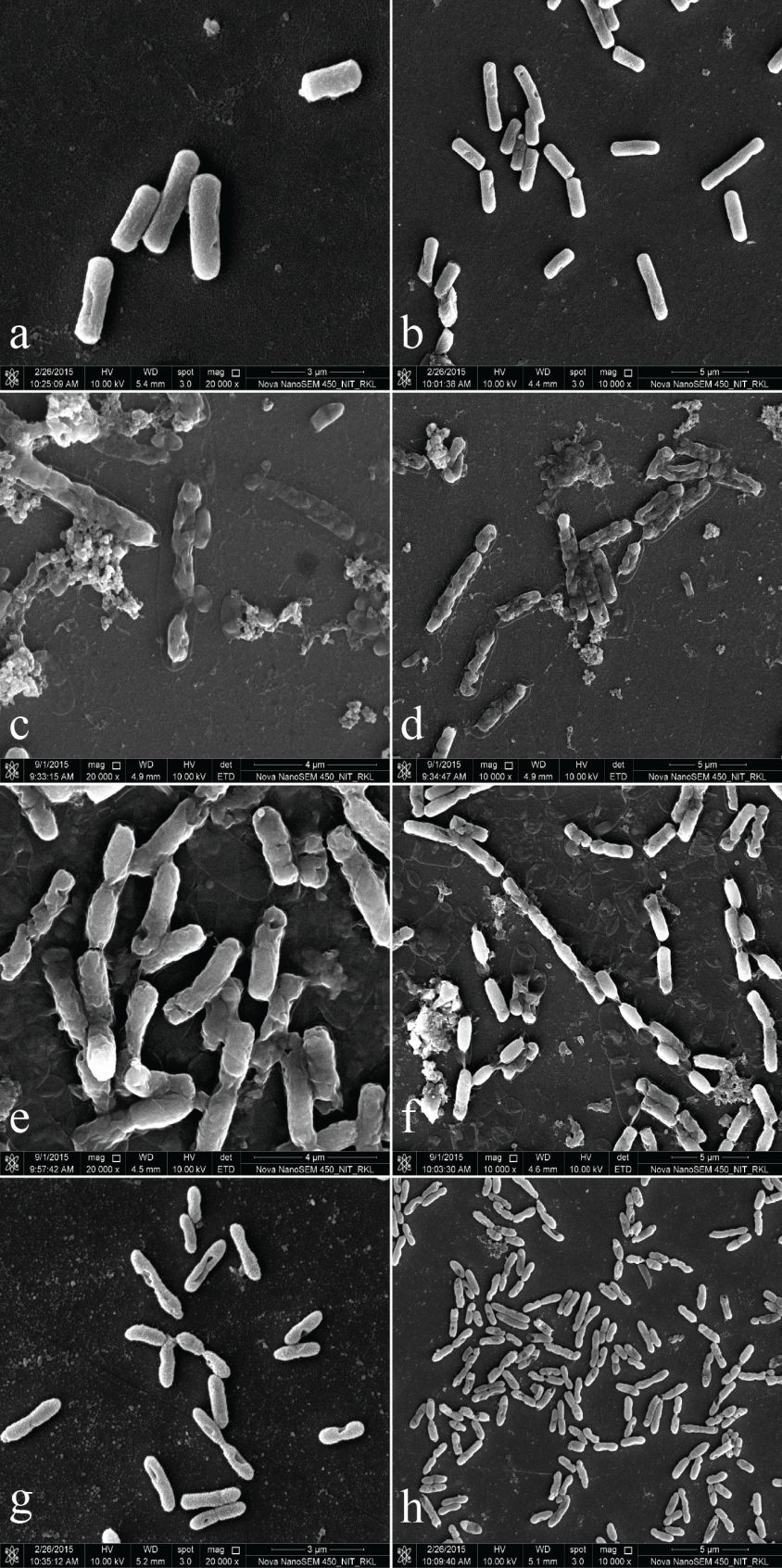
**

**Supplementary Fig. S7. Scanning electron micrographs showing the surface morphology of *B. subtilis* in different growth phases treated with Nisin.** (a, b) Untreated bacteria morphology of early stationary phase. All rest of the images is bacteria taken from different growth phases post-nisin treatment, (c, d) bacterial morphology in late lag phase, (e, f) bacterial morphology in mid-log phase, and (g, h) bacterial morphology in early stationary phase. (left - high magnification), right - low magnification))

**Supplementary Fig. S8**


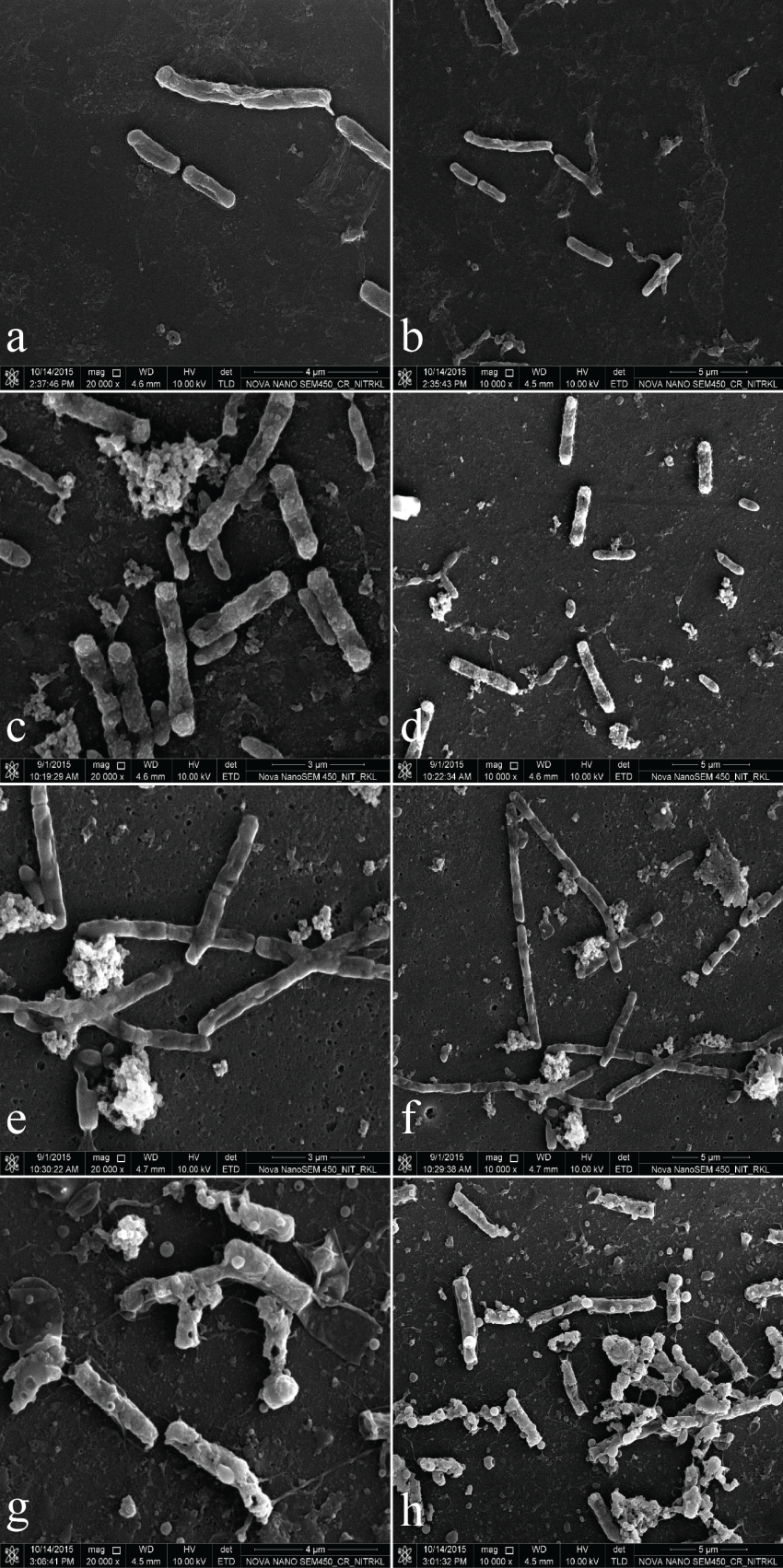


**Supplementary Fig. S8. Visualization of *E. coli* surface morphology with scanning electron microscope in different growth phases treated with Nisin.** (a, b) Untreated bacterial morphology of early stationary phase. All rest of the images is bacteria taken from different growth phase post-nisin treatment, (c, d) bacterial morphology in late lag phase, (e, f) bacterial morphology in mid-log phase, and (g, h) bacterial morphology in early stationary phase. (left - high magnification, right - low magnification)

**Supplementary Fig. S9**


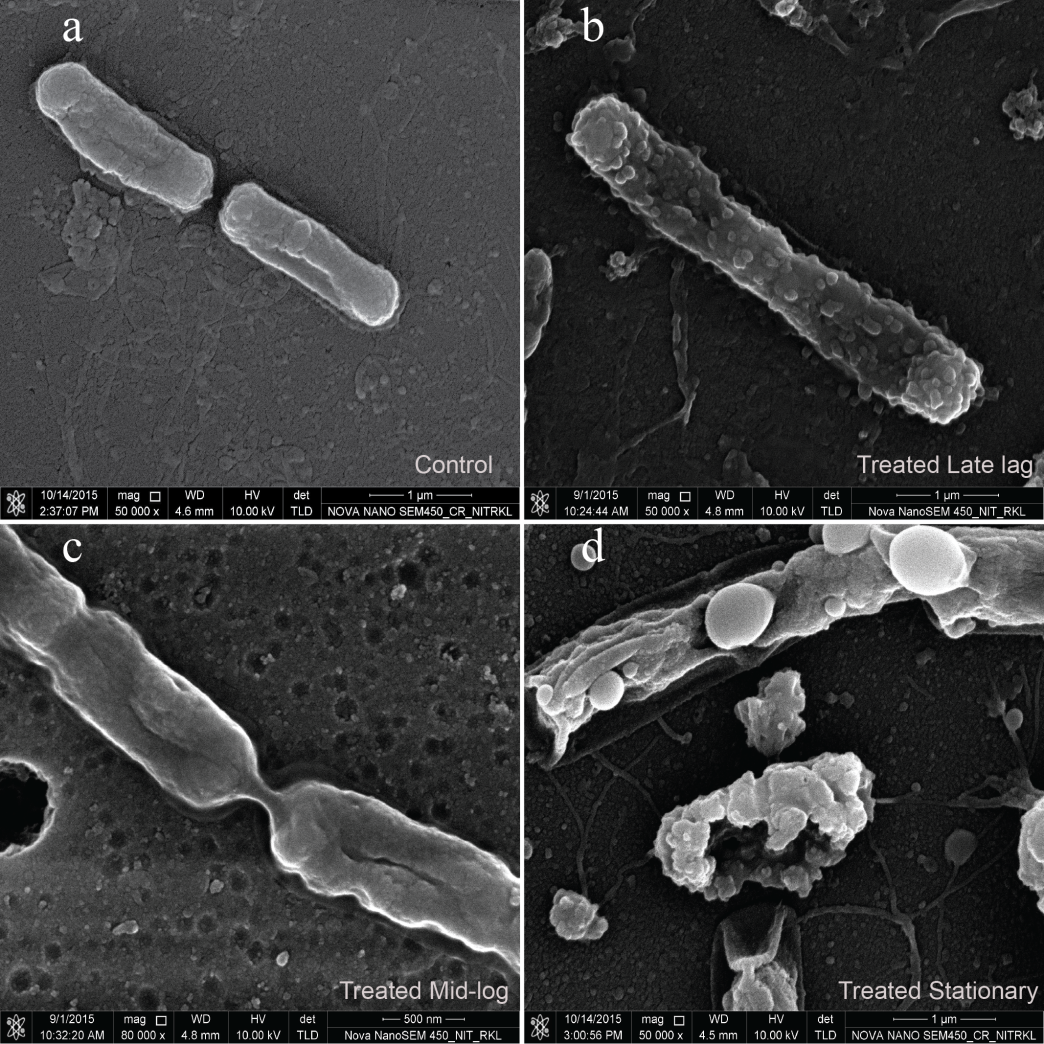


**Supplementary Fig. S9. High-resolution SEM images of *E. coli* treated with nisin.** (a) Untreated bacterial morphology seen in stationary phase. After nisin treatment - (b) late lag (c) mid-log and (d) stationary phase.

**Supplementary Fig. S10**

**
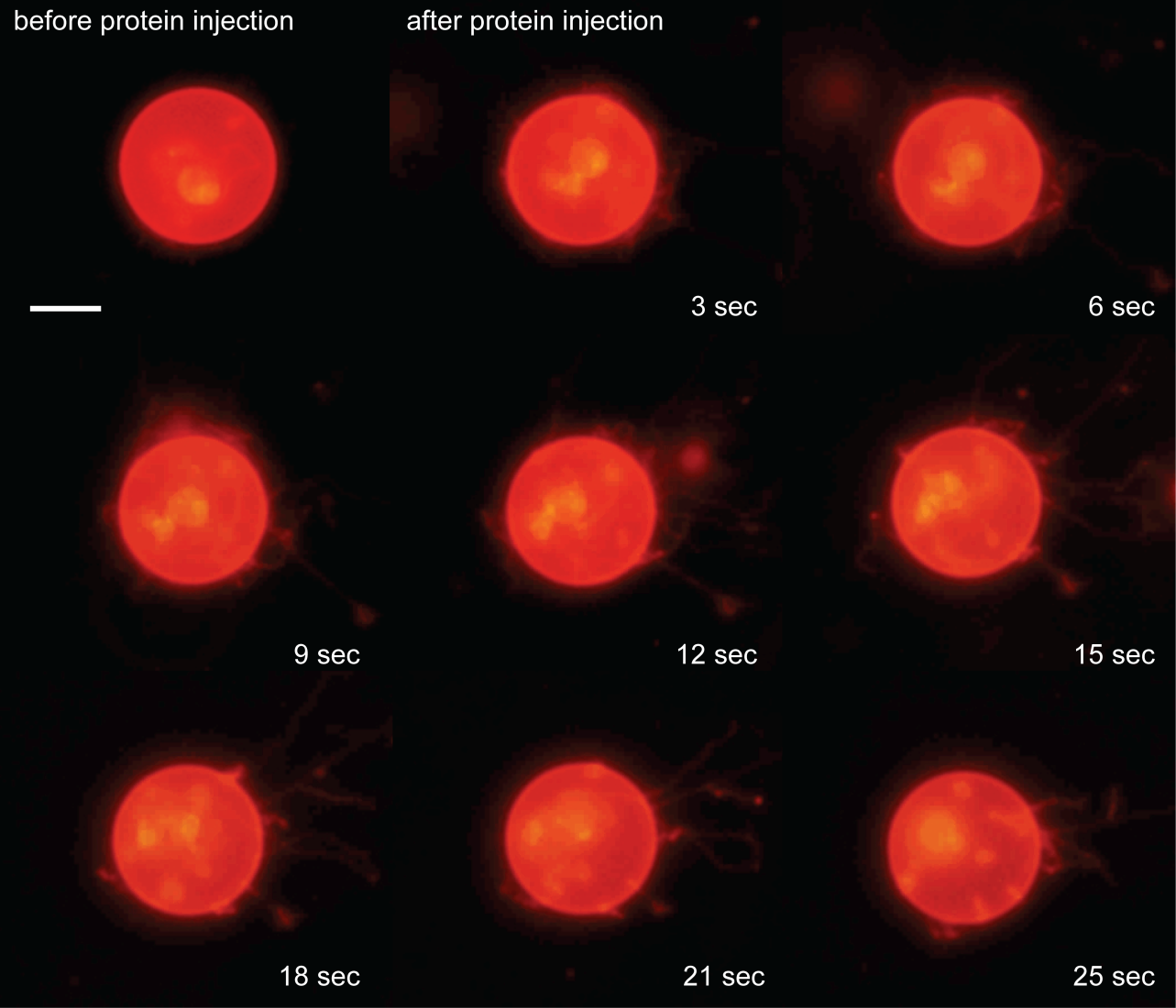
**

**Supplementary Fig. S10. Time-lapse fluorescence images showing Nisin (*unlabeled*) induced tubulation in giant liposome made up of zwitterionic DOPC (*red*, doped with Rhodamine PE). Scale bar, 5 µm.**

**Supplementary Fig. S11.**

**
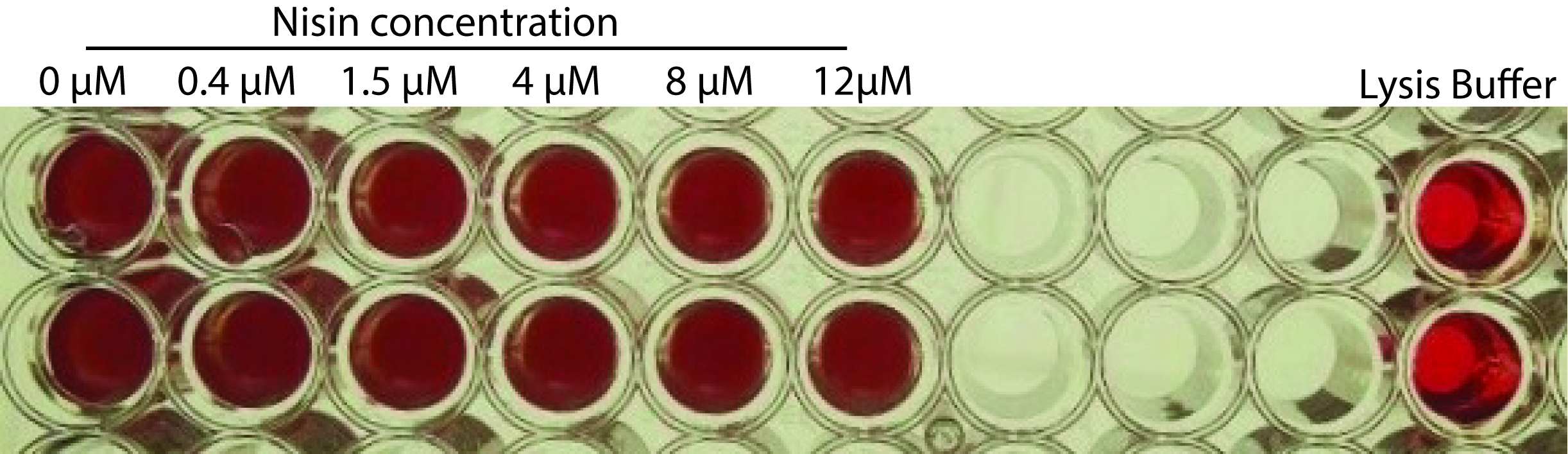
**

|  | **Absorbance (595 nm)** | | | | | | |
| --- | --- | --- | --- | --- | --- | --- | --- |
| **Nisin ( µM)** | **0** | **0.4** | **1.5** | **4** | **8** | **12** | **Lysis buffer** |
| **Sample 1** | 2.84 | 2.80 | 2.78 | 2.84 | 3.09 | 3.16 | 0.92 |
| **Sample 2** | 2.75 | 2.74 | 2.82 | 2.82 | 3.01 | 3.23 | 0.98 |

**Supplementary Fig. S11. Image of microplate wells showing the effect of nisin on RBC demonstrated by haemolytic assay**. RBC are treated with increasing concentrations of nisin i.e. 0.4, 1.5, 4, 8, and 12 µM from left to right in duplicate. Positive control was taken as treatment of RBC with lysis buffer. Absorbance for corresponding samples at 595 nm was recorded which clearly suggests presence of intact RBCs that have settled down and thus giving rise to higher absorbance. On the contrary, RBC treated with lysis buffer causes rupture of RBC and release of cytosolic components into the surrounding thus giving rise to a relatively more homogenous/transparent appearance and thus lower absorbance.

**Supplementary Table S1. Surface roughness profile of nisin-treated bacteria.**

| **Nisin (µM)** | **Surface Roughness** | |
| --- | --- | --- |
|  | ***B. subtilis*** | ***E. coli*** |
| 0 | 73.58 ± 2.58 | 57.78 ± 3.85 |
| 0.4 | 77.84 ± 3.59 | 59.82 ±4.26 |
| 12 | 85.43 ± 4.19 | 69.16 ±3.64 |

**Supplementary Table S2. Surface roughness profile of nisin-treated bacteria during different phase of growth.**

| **Growth Phase** | **Surface Roughness** | |
| --- | --- | --- |
|  | ***B. subtilis*** | ***E. coli*** |
| Control | 73.58 ± 2.58 | 57.78 ± 3.85 |
| Treated Late Lag | 83.19 ± 5 | 68.9 ± 3.99 |
| Treated Mid-log | 95.66 ± 1.13 | 72.29 ± 2.4 |
| Treated Stationary | 85.43 ± 4.19 | 69.16 ±3.64 |

**Supplementary Table S3. Effect of nisin treatment on morphological features of *B. subtilis.*** Dimensional analysis of the changes in length, breadth, surface area and an approximate estimate of lipids for *B. subtilis* in early stationary phase. Mean ± SE, skewness and kurtosis of the data are shown. Skewness values close to zero and kurtosis values close to 3 suggest a normal distribution pattern in the observations made. (n = 51)

| ***B. subtilis*** | **Length (µm)** | | | **Breadth (µm)** | | | **Surface Area (µm^2^)** | | | **No. of Lipids** | | |
| --- | --- | --- | --- | --- | --- | --- | --- | --- | --- | --- | --- | --- |
| **nisin (µM)** | Mean | Skewness | Kurtosis | Mean | Skewness | Kurtosis | Mean | Skewness | Kurtosis | Mean | Skewness | Kurtosis |
| **0** | 2.56 ± 0.055 | 0.644 | 2.4 | 0.88 ± 0.011 | -0.207 | 2.7 | 8.26 ± 0.202 | 0.619 | 2.5 | 1.65 x 10^7^ ± 4.04x10^5^ | 0.619 | 2.1 |
| **0.4** | 2.24 ± 0.053 | -0.012 | 3.1 | 0.85 ± 0.016 | -0.125 | 2.2 | 7.17 ± 0.212 | 0.301 | 2.6 | 1.43 x 10^7^ ± 4.24x10^5^ | 0.301 | 2.6 |
| **12** | 1.17 ± 0.029 | 0.331 | 2.7 | 0.38 ± 0.009 | 0.627 | 2.8 | 1.63 ± 0.059 | 0.251 | 2.4 | 3.27 x 10^6^ ± 1.19x10^5^ | 0.251 | 2.5 |
